# An ethnobotanical analysis of parasitic plants (*Parijibi*) in the Nepal Himalaya

**DOI:** 10.1186/s13002-016-0086-y

**Published:** 2016-02-24

**Authors:** Alexander Robert O’Neill, Santosh Kumar Rana

**Affiliations:** Fulbright-Nehru Research Scholar, G. B. Pant Institute of Himalayan Environment & Development, Gangtok, East Sikkim India; Department of Botany, Systematics and Biodiversity, Central Department of Botany, Tribhuvan University, Kirtipur, Kathmandu 44618 Nepal

**Keywords:** Nepal, Himalaya, Ethnobotany, Parasitic plants

## Abstract

**Background:**

Indigenous biocultural knowledge is a vital part of Nepalese environmental management strategies; however, much of it may soon be lost given Nepal’s rapidly changing socio-ecological climate. This is particularly true for knowledge surrounding parasitic and mycoheterotrophic plant species, which are well represented throughout the Central-Eastern Himalayas but lack a collated record. Our study addresses this disparity by analyzing parasitic and mycoheterotrophic plant species diversity in Nepal as well as the ethnobotanical knowledge that surrounds them.

**Methods:**

Botanical texts, online databases, and herbarium records were reviewed to create an authoritative compendium of parasitic and mycoheterotrophic plant species native or naturalized to the Nepal Central-Eastern Himalaya. Semi-structured interviews were then conducted with 141 informants to better understand the biocultural context of these species, emphasizing ethnobotanical uses, in 12 districts of Central-Eastern Nepal.

**Results:**

Nepal is a hotspot of botanical diversity, housing 15 families and 29 genera of plants that exhibit parasitic or mycoheterotrophic habit. Over 150 of the known 4500 parasitic plant species (~3 %) and 28 of the 160 mycoheterotrophic species (~18 %) are native or naturalized to Nepal; 13 of our surveyed parasitic species are endemic. Of all species documented, approximately 17 % of parasitic and 7 % of mycoheterotrophic plants have ethnobotanical uses as medicine (41 %), fodder (23 %), food (17 %), ritual objects (11 %), or material (8 %).

**Conclusions:**

Parasitic and mycoheterotrophic plant species exhibit high diversity in the Nepal Central-Eastern Himalaya and are the fodder for biocultural relationships that may help inform future environmental management projects in the region.

**Electronic supplementary material:**

The online version of this article (doi:10.1186/s13002-016-0086-y) contains supplementary material, which is available to authorized users.

## Background

Indigenous biocultural knowledge (IBK) is pillar of environmental management strategies in Nepal, and has been adopted into policies that attempt to ensure that indigenous communities live in and benefit from ‘nature’ in a sustainable manner. For over two decades, IBK-conscious legislation such as the Forest Act [1], Forest Regulation Act [2], and National Biodiversity Strategy [3] as well as international contracts with the Convention on International Trade in Endangered Species of Wild Flora and Fauna [4], Ramsar Convention [5], and United Nations Convention on Biological Diversity [6] have cultivated cooperative relationships between Indigenous and local groups and management officials. Today, over 35 % of the 27.8 million-person population participates in Nepal’s vibrant community forestry program [7, 8], with over 70 % of the total population directly dependent on wild-forest crops for primary livelihood, food, and medicine [9]. IBK-conscious polices have bolstered existing socio-ecological relationships in Nepal, conserved natural resources, and preserved the country’s cultural heritage.

However, in spite of recent successes, Nepal’s current policies face impending challenges from ‘modernizing’ forces and accelerated rates of environmental change [10]. For instance, population growth, human migration, and agricultural development have had pernicious ramifications in many sacred and protected zones, including Chitwan National Park [11–13]. At higher altitudes, overharvest of medicinal plants, driven primarily by market demands in India and China [14], has disrupted historical ecosystem dynamics and transformed traditional livelihoods [15, 16]. Beyond these acute sources of environmental degradation, trends in migrant labor and education have further stunted rates of IBK transmission, reducing the practicality of existing policies [17]. Therefore, future conservation strategies must, in part, preserve IBK that may provide human and ecological communities with greater adaptive capacity to cope with current and future environmental change.

IBK in the form of Traditional Botanical Knowledge (TBK) may provide the most viable option for ameliorating current rates of biocultural attrition in Nepal. TBK incorporates perceptions of natural environments, including elements such as soil, climate, vegetation type, stages of ecological succession, and land use [15], and has been celebrated for its ability to support local economies through alternative livelihoods [16]. Nepal ranks as the 9th most floristically diverse country in Asia. Despite occupying 0.1 % of earth’s land cover, it houses over 8000 plant species of which one quarter are believed endemic [10, 18]. Approximately 50 % of these plants are considered ‘useful’ [19] or ‘ethnobotanical’ in nature [20] and 25–50 % are expected to have medicinal properties [19, 21, 22]. Agroforestry and sustainable harvesting practices of medicinal or useful plant species, including many culinary species such as cardamom, may provide the economic incentive [23, 24] necessary to ensure the future health of Nepalese ecosystems [25].

Although there has been a recent surge in TBK research, certain species remain significantly understudied in Nepal. In particular, the guild of plants known as parasites and mycoheterotrophs, collectively denoted by the term *parijibi* in Nepali language, lacks a literature record. Parasites and mycoheterotrophs (PMP) are unique among plants because they depend upon a host plant for some or all of their nutrients during a period of their lifecycle. Globally, there are 20 parasitic plant families and eight mycoheterotrophic plant families, many of which do not photosynthesize, and, therefore, have atypical life histories. Moreover, all PMPs have extreme habitat specifications that are inherently bound to forest community dynamics as well as their host-species ranges. Together, these requirements have had marked affects on PMP population densities, abundances, and potential ranges. PMP are well represented in Nepal; however, little is known about their exact diversity or the biocultural knowledge that surrounds them.

To address this disparity, our study aims to create the first compendium of PMP taxa in Nepal, including their growth habit, geographic distribution, altitudinal range, host plants, flowering and fruiting times. We then seek to create a comprehensive biocultural record of PMPs, emphasizing TBK and ethnobotanical uses, to preserve the biocultural heritage of these species in the Central-Eastern Himalayas. Based on fieldwork conducted from September 2013 through May 2014 and an exhaustive literature review, we developed a critical interpretation of PMP use and management.

## Methods

### Study area

Nepal occupies a 147,181-km^2^ zone in the Central-Eastern Himalayan range (latitude: 26°22′ to 30°27′ N; longitude: 80°40′ to 88°12′ E) between China and India. It is administratively divided into five development regions, 14 zones, 75 districts, 191 municipalities, and 3276 village development committees (VDC). At the level of VDC, most communities are further subdivided along ethnic or caste lines, stratifying IBK/TBK well beyond the level of administrative boundaries.

**Fig. 1:**
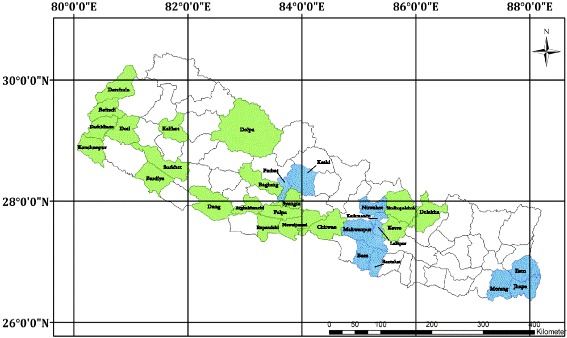
Map of the Nepal Central Himalaya. *Blue*: Districts surveyed during our botanical and ethnobotanical fieldwork. *Green*: Districts where previous reports detailed the ethnobotanical uses of parasitic plants

Ecologically, the country is classified into three vegetative and seven physiogeographic zones based on altitudinal variations from the lowlands (59 m) to the high Himalayas (8848 m). However for the purposes of policy, the Master Forestry Plan for Nepal considers only five physiogeographic zones based on altitude: Terai (60–330 m), Siwalks (120–2000 m), Middle Mountain (2000–3000 m), High Mountains (3000–4000 m) and High Himal (above 4000 m). Our fieldwork was primarily conducted in the Terai and Middle Mountains of Central and Eastern Nepal. Our review spans the entire country (Fig. 1).

### Ethnobotanical survey

Before initiating our ethnobotanical investigation, we collected all available data on the status of PMP in Nepal. First, we reviewed authoritative botanical texts [25–30] to glean details on plant distributions, altitudinal ranges, parasitic habit, hosts, flowering times, and fruiting times. We then cross-referenced these data against online botanical databases [31–33], and compared these data against herbaria records at Nepal’s National Herbarium and Plant Laboratories (KATH) [34] in Godawari, Lalitpur, Tribhuvan National University’s Central Department of Botany’s Herbarium (TUCH) [35] located in Kirtipur, Kathmandu, and Tribhuvan University Post-Graduate Campus’ botanical collections in Biratnagar, Morang (TUCH; 34). Once this process was complete, we generated range maps and species profiles for each PMP using ArcGIS version 9.3 to guide our ethnobotanical survey [36] (Additional files 1, 2 and 3). A linear regression analysis was then performed to understand how altitudinal gradients correlate with PMP diversity in Nepal.

**Fig. 2:**
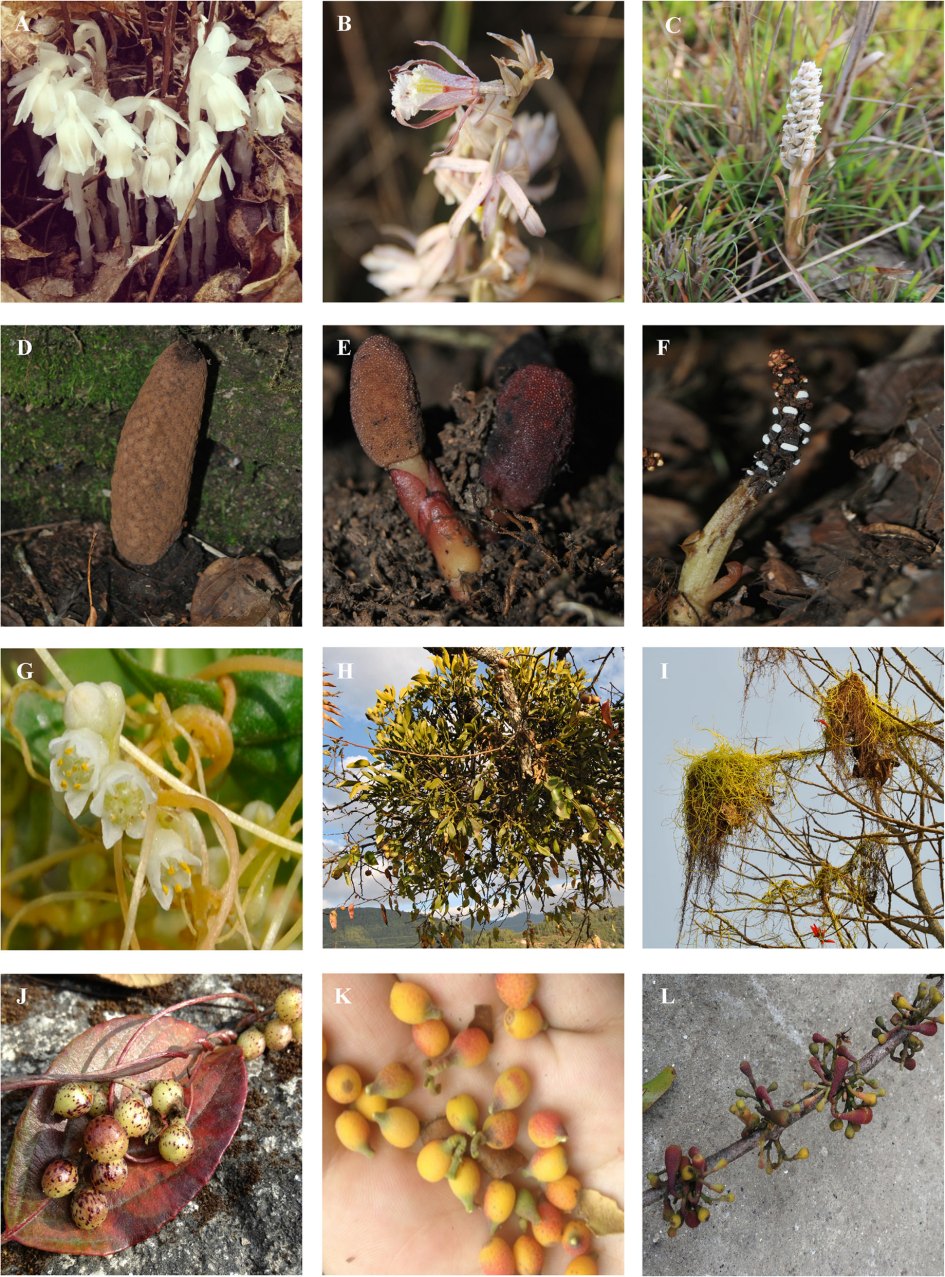
Photographic record of some parasitic and mycoheterotrophic plants documented during our study. Panels **a**–**c**: Mycoheterotrophic plants native to Nepal, including *Montropa uniflora* and two orchid species found in Chitwan National Park known by the Tharu term *chikhtaa*. Panels **d**–**f**: Growth habit of *prumai*, or species in the Balanphoraceae (Panel **d**: Fruiting body of *Rhopalocnemis phalloides*: Panels **e** and **f**: Female and male inflorescence of *Balanophora polyandra*, respectively). Panels **g**–**i**: Growth habit of *Cuscuta chinensis*, *Viscum album*, and *Cuscuta reflexa*. Panels **j**–**l**: Different parasitic plant fruit consumed by our informants, including *Cuscuta* and two mistletoe species (Loranthaceae)

Once botanical data were collated, we conducted field expeditions to evaluate the presence and perceptions of PMP in 12 districts based on high levels of reported biological and cultural diversity: Bara, Chitwan, Ilam, Jhapa, Kathmandu, Kaski, Lalitpur, Makwanpur, Morang, Nuwakot, Parbat, and Rautahat Districts (Table 1). Some larger VDCs visited during this time include: Akumba (Bara), Biratnagar (Morang), Birtamode (Jhapa), Chitre (Kaski), Daman (Makwanpur), Mhanegang (Nuwakot), and Sikles (Kaski). At each site, we surveyed ecosystems with the help of local guides in order to evaluate the presence of PMPs at each site. When permitted, we collected samples for use during interviews. Informants were later presented with freshly pressed or gathered plant material; in some cases, dried specimens or high-resolution, color photographs were used due to harvesting regulations (e.g. Fig. 2).

**Table 1:** Parasitic plant families represented in Nepal. See Additional file 1 for species-level profiles and Additional file 3 for species range maps

Family	Genus	Number of species
Amphorogynaceae	*Dufrenoya*	2
Balanophoraceae	*Balanophora*	3
	*Rhopalocnemis*	1
Cervantesiaceae	*Pyrularia*	1
Convolvulaceae	*Cuscuta*	4
Loranthaceae	*Dendrophthoe*	2
	*Helixanthera*	2
	*Loranthus*	2
	*Macrosolen*	1
	*Scurrula*	4
	*Taxillus*	2
Olaceae	*Olax*	1
	*Erythropalum*	1
Opiliaceae	*Cansjera*	1
	*Lepionurus*	1
Orobanchaceae	*Aeginetia*	2
	*Boschniakia*	1
	*Buchnera*	2
	*Centranthera*	2
	*Euphrasia*	7
	*Leptorhabdos*	1
	*Orobanche*	6
	*Pedicularis*	71
	*Phtheirospermum*	1
	*Striga*	4
Santalaceae	*Osyris*	2
	*Thesium*	2
	*Santalum*	1
Schoepfiaceae	*Schoepfia*	1
Viscaceae	*Viscum*	5

At each study site, we interviewed both specialist and non-specialist plant user groups using a Rapid Rural Appraisal method [37]. Specialist groups reviewed and consulted during this time include traditional doctors or healers known variously as *amchis*, *bonpos*, *dhamis*, *jhankris*, and *khabres* as well as management officials, such as plant quarantine officers and junior technical agricultural assistants. Non-specialist groups consisted of people, including farmers, trade workers, and students, for whom plants are not an important component of their professional life, but who may use them for other purposes or personal use [16]. We spoke with all who readily accepted to be interviewed; however, we took care to involve no more than one informant from the same household during a single interview session.

Using an informal, semi-structured questionnaire (Additional file 4), we evaluated biocultural knowledge in terms of the informant’s ability to identify PMPs and describe their ethnobotanical uses. They were then asked if they recognized the plant, if there was a local name for the plant, if it had any uses, and if they personally used it [16]. We also asked general questions related to population abundance, including population distributions, localities, habitat types, and characteristics of different populations. More specific questions focused on knowledge of the biology and ecology of PMPs, including their life histories. Before each interview, prior informed consent was obtained with the help of district-level and village-level community leaders, government officials, and local guides to collect and disseminate their IBK (Cornell University IRB: 1311004259).

In total, we interviewed 141people (Male: 89, Female: 52; Average Age: 51 years) from both specialist and non-specialist groups (Table 1). Informants varied in ethnic identity, including Brahmin/Chhetri (14 %), Dalit (10 %), Gurung (27 %), Madeshi/Tharu (17 %), Rai (8 %), and Tamang (24 %). Approximately 62 % of those interviewed had no formal education, and approximately 72 % engaged in agricultural or pastoral livelihoods. All informants had lived in their respective village for their entire lives with the exception of five Gurung men in Kaski District who served for an average of 20 years each in the Indian Army or British Army’s Gurkha regiment. Use accumulation curves were used to determine the number of interviews conducted for each PMP per site was sufficient [37].

When permitted, herbaria specimens were also collected and voucher records mounted following standard procedures [16]. Most specimens were identified to the species level and were deposited at TUCH in Kirtipur, Kathmandu. In addition, we took photographs and recorded species information, geographical coordinates, altitude, and habitat type and characteristics on herbaria records as well as in Additional files 1, 2 and 3. Finally, R. P. Chaudhary of Tribhuvan University’s RECAST Division, as well as P. K. Jha and K. K. Shrestha of Tribhuvan University’s Central Department were consulted regarding species identification and study methods.

We then conducted an exhaustive literature review on the ethnobotanical uses and biocultural knowledge surrounding parasitic and mycoheterotrphic species to supplement our field research [19, 26, 29, 38–86]. Our review targeted data on common names, plant uses, and plant preparations. Our internet surveys were conducted using the study country’s name (Nepal), plant species’ name, and the following keyword combinations: ethnobiology, ethnobotany, ethnoecology, ethnopharmacology, ethnobiological, ethnobotanical, ethnoecological, ethnopharmacological, and ethnoveterinary. We also visited the Tribhuvan University and Cornell-Nepal Study Program libraries (Kirtipur, Kathmandu) to collect all available information from unpublished Master’s thesis. We are aware that our collection criteria, although exhaustive, did not include all unpublished studies and/or all local journals or articles not published in English, Nepali, or Tibetan languages.

## Results and discussion

### Parasitic and mycoheterotrophic plant diversity

**Fig. 3:**
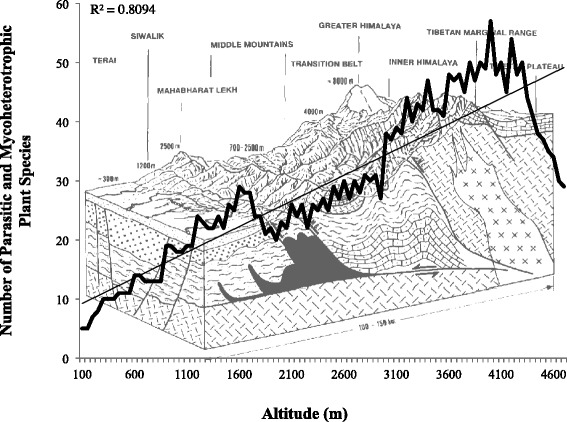
Total number of parasitic and mycoheterotrophic plant species found along Nepal’s altitudinal gradient. Parasitic and mycoheterotrophic species diversity is highly correlated with altitude (R^2^ = 0.8094), with greater species richness found in high-altitude zones (background image: [88])

Nepal is a hotspot for PMP diversity. Botanical records revealed that 150 of the Earth’s 4500 parasitic plant species (3 %; Table 2) and 28 of the approximately 160 mycoheterotrophic species (18 %; Table 3) are native to Nepal (Additional files 1 and 2). Many of the records parasitic species are also considered Nepal endemic, including: *Euphrasia nepalensis*, *Pedicularis annapurnensis*, *Pedicularis anserantha*, *Pedicularis brevicaposa*, *Pedicularis chamissonoides*, *Pedicularis cornigera*, *Pedicularis mugensis*, *Pedicularis odontolma*, *Pedicularis oxyrhyncha*, *Pedicularis pseudoregeliana*, *Pedicularis tamurensis*, *Pedicularis terrenoflora*, *Pedicularis yalungensis*. Altitude and number of PMPs are strongly correlated (R^2^ = 0.81), with higher altitudes exhibiting greater PMP species richness (Fig. 3). This correlation is primarily driven by parasitic *Pedicularis* spp. found at high altitudes.

**Table 2:** Fully mycoheterotrophic plant families represented in Nepal. See Additional file 2 for species-level profiles and Additional file 3 for species range maps

Family	Genus	Number of species
Burmanniaceae	*Burmannia*	2
Ericaceae	*Monotropa*	2
	*Monotropastrum*	1
Gentianaceae	*Exacum*	1
Orchidaceae	*Eulophia*	6
	*Galeola*	1
	*Neottia*	2
	*Cephanlanthera*	1
	*Epipogium*	2

**Table 3:** Details on informants surveyed by our study according to profession, age, and sex/gender in each district surveyed in our study

District	Types of users	Major profession(s)	Number of informants	Sex/Gender	Age range
Bara	Non-specialist	Agro-pastoralists/Carpenters	11	6 Male	30–85
				5 Female	20–55
	Specialist	Junior Technical Agricultural Assistants	2	2 Male	25–35
Chitwan	Non-specialized	Eco-tourist Guides/Hotel Owners	7	5 Male	20–40
				2 Female	
	Specialist	Park Officials	3	3 Male	20–35
Jhapa	Non-specialist	Agro-pastoralists/Merchants/Students	19	14 Male	20–80
				5 Female	25–50
	Specialist Users	Agro-pastoralist/*Jhankri*	1	1 Male	36
Kaski	Non-specialist	Agro-pastoralists/Students	45	25 Male	20–70
				20 Female	
	Specialist	*Jhankri*/*Kabre*	7	5 Male	60–85
		Park Officials		2 Male	30–40
Makwanpur	Non-specialist Users	Agro-pastoralists/Carpenters/Hotel Owners	14	6 Male	40–70
				8 Female	
Morang	Non-specialist	Sugarcane Harvesters	5	5 Male	30–60
Nuwakot	Non-specialist	Agro-pastoralists	17	8 Male	35–65
				9 Female	
	Specialist	*Dhami*/*Bonpo*	2	2 Male	45–60
Rautahat	Non-specialist	Agro-pastoralists	8	5 Male	40–70
				3 Female	30–50

Our ethnobotanical survey documented the uses of 15 species and varieties of Mistletoe (Loranthaceae: *Dendropthoe falcata*, *D. pentandra*, *Helixanthera ligustrina*, *H. parasitica*, *Loranthus odoratus*, *Macrosolen cochinchinensis*, *Scurrula elata*, *S. parasitica*, *S. pulverenta*, *Taxillus umbellifer*, *T. vestitus*; Viscaceae: *Viscum album*, *V. articulatum* var. *articulatum*, *V. articulatum* var. *liquidambariclum*), four species in the Orobanchaceae (*Aeginetia indica*, *Orobanche aegyptiaca*, *Striga angustifolia*, *S. gesnerioides*), three species in the Convolvulaceae (*Cuscuta chinensis*, *C. europaea* var*. indica*, *C. reflexa* var. *reflexa*), two species in the Balanophoraceae (*Balanophora polyandra*, *Rhopalocnemis phalloides*), two species in the Orchidaceae (Fig. 2), and one species in the Ericaceae (*Monotropa uniflora*). We created 42 herbarium records for 21 species of PMP, which were deposited at TUCH (Additional file 5). Our survey is the first to record the following plants per district: *Balanophora polyandra* (Kaski), *Cuscuta chinensis* (Jhapa), *C. reflexa* (Bara, Jhapa, Rautahat); *Dendropthoe falcata* (Morang); *D. falcata* (Rautahat); *Helixanthera parasitica* (Makwanpur); *Macrosolen cochinchinensis* (Jhapa); *Rhopalocnemis phalloides* (Kaski).

### Ethnobotanical uses of parasitic and mycoheterotrophic plants (Parijibi)

Ethnobotanical information for 23 parasitic plant species distributed among four families and 13 genera were documented during our fieldwork (Table 4). Approximately 82 % of informants surveyed were familiar with all PMP species native or naturalized to their local environment, and their uses generalized into five categories: medicine (41 %), fodder (23 %), food (17 %), ritual objects (11 %), or material (8 %). The largest proportion of ethnobotanicals emerged from the Loranthaceae and Convolvulaceae. Our literature review revealed previous ethnobotanical records for 10 parasitic and two mycoheterotrophic plant species not covered by our survey, with the majority of species in the Orobanchaceae (*Pedicularis* spp.). *Pedicularis* spp. were primarily utilized by Tibetan groups living in high-altitude regions of the Central Himalayas, which corresponds with our species diversity data (Fig. 3). Based on studies in the 29 total districts surveyed, approximately 17 % of all parasitic plants and 7 % of all mycoheterotrophic plants native to Nepal have ethnobotanical uses. Our survey provides the first ethnobotanical data on *Balanophora polyandra*, *Cuscuta chinensis*, and *Rhopalocnemis phalloides* in Nepal.

**Table 4:** Ethnobotanical uses of parasitic and mycoheterotrophic plants in the Nepal Central Himalaya

Scientific name and voucher number(s)^a^	Vernacular name(s)^b^	Part(s) used	Traditional use(s)	Reference(s)	Notes on ethnobotanical use(s)
*Aeginetia indica* L.	*Ankuri Makuri* ^NP^, *Kum Kum* ^NP^, *Puksur* ^NP^,	Entire Plant	Ritual Object	Current Study	The entire plant is placed in shrines or on alters during Teej festival as a symbol of Shiva and Parvati.
	*Gaura Parbata* ^NP^		Medicine	[26, 89]	As medicine, the fresh plant juice is consumed to reduce fever.
*Balanophora polyandra* Griffith.	*Ek Lebir* ^NP^	Entire Plant	Ritual Object	Current Study	Both *jhankris* and *kabres* that *R. phalloides* exhibits particularly potent spiritual properties; however, *B. polyandra* is also used for a variety of ritual purposes. As a ritual object, both plants are collected on Tuesdays, decorated with turmeric, and kept inside the house. They may be combined with *Citrus* spp. to combat the evil eye or to ward off spirits. As medicine, the root of *B. polyandra* is dipped in hot/boiling water and then massaged on the afflicted area. For use as vermicide, the entire plant is ground into paste and diluted, and then consumed for a week.
Rana ARO 41			Medicine		
*Rhopalocnemis phalloides* Jungh.	*Ek Lebir* ^NP^	Entire Plant	Ritual Object	Current Study	
Rana ARO 42					
*Boschniakia himalaica* Hook. & Thomson ex Hook.	*Besegano* ^NP^, *Kangdol* ^TA^	Entire Plant	Ritual Object	Current Study	The entire plant is placed in shrines or on alters during various festivals, including Teej. The festivals and the blooming time for this species allign.
				[29]	
*Centranthera cochinchinensis* var. *nepalensis* (D. Don) Merr.	*Gumteolee* ^NP^	Entire Plant	Fodder	[29]	The entire plant is an alternative fodder.
*Cuscuta chinensis* Lam.	*Aakashjeli* ^NP^,Dul-shag^TI^, *Ghu-ghu-sazin* ^TI^	Entire Plant	Medicine	Current Study	People do not differentiate the use of *Cuscuta* at the level of the taxonomic classification.
O’Neill Rana ARO 2			Ritual Object	[81]	Instead, yellow color is the only essential factor considered when harvesting *Cuscuta* tendrils. As medicine, *Cuscuta* is used to treat heptatic diseases, including jaundice. Fresh tendrils are washed and ground into paste.
*Cuscuta europaea* var. *indica* Engelm.	*Aakashbeli* ^NP^, *Drhul-shuck* ^TI^, *Mhasu Lahara* ^NW^,	Entire Plant	Medicine	Current Study,	This paste is then mixed with hot water and consumed as a soup (*jhol*) for as long as symptoms persist.
O’Neill Rana ARO 1, 18	*Sati* ^NP^		Fodder	[29, 80, 90]	Variations on this treatment include boiling fresh plants and then inhaling the vapor, or placing *Cuscuta* tendrils under the patients’ bed to enhance the recovery process. This preparation, particularly as soup, is also used and consumed to treat asthma, body pains, cough, dandruff, diarrhea, gastric pain, headache, stomach disorders, tonsilitis, and urticaria. Some practitioners expose patients’ bodies to plant smoke to reduce swelling. Tibetan groups perscribe this plant to treat reproductive disorder and to increase libido or sex drive.
*Cuscuta reflexa* var. *brachystigma* Englem.	*Aakashbeli* ^NP^, *Aakashlati* ^TH^,	Entire Plant	Medicine	Current Study	
O’Neill Rana ARO 39	*Amar Lata* ^NP^, *Amarvel* ^NP^, *Baora* ^TH^		Fodder	[91]	
*Cuscuta reflexa* var. *reflexa* Roxb.	*Aakashbeli* ^NP^, A*kasbela* ^NP^, *Akasbeli* ^NP^, *Akasebeli* ^RJ^,	Entire Plant	Medicine	Current Sudy	*Cuscuta* is also invoked during healing rituals in the Terai as a symbol of Shiva’s hair. In these locations, fresh tendrils are also worn as a protective amulet. Our study also recorded two ethnoveterinary uses in Tamang communities, including as a poultice to treat wounds and as a tonic relieving blood from the urine of bulls (loombhadi). As fodder, only red tendrils are consumed as they are considered to be less bitter.
O’Neill Rana ARO 9, 10, 12, 13, 19	*Akashbel* ^NP^, *Akashbeli* ^NP^, *Akashabeli* ^NP^, *Aakashjeli* ^GU^,		Fodder	[19, 29, 38–59, 90]	
	*Akashe Lahara* ^MA^, *Amar Lata* ^NP/LI^, *Amarlathi* ^TH^,		Ritual Object		
	*Amaruela* ^SN^, *Asparsa* ^SN^, *Baora* ^TH^,				
	*Bimfang-gummu-bidong* ^ME^, *Chimchimpona* ^LI^,				
	*Chhoti Hadjori* ^TH^, *Janailaharo* ^NP^, *Piyari* ^TH^,				
	*Sewanli* ^TH^, *Taarghey* ^TA^, *Ur-lang-du* ^TA^, Urlara^TA^				
*Dendrophthoe falcata* (L.f.) Etting.	*Ainjeru* ^NP^, *Ajeru* ^NP^, *Banda* ^NP^,*Rhiniya* ^MO/NP^,	Aerial Parts	Medicine	Current Study	Practitioners grind leaves into a paste to treat dermic conditions, including rashes, pus, and boils.
O’Neill Rana ARO 14	*Mandargon Banda* ^SA^, *Nihi* ^TA^	Fruit	Food	[29, 41, 60–69]	Pulverated bark paste is also used as an abortifacient and to correct menstural problems. When combined with other plants, the paste can be used to treat fractures. Children consume its sweet fruit, which is also considered to be antiseptic. Leaves may be combined with *Urtica doica* to treat bone fractures.
*Dendrophthoe pentandra* (L.) Miquel.	*Ainjeru* ^NP^	Entire Plant	Fodder	Current Study	The entire plant is an alternative fodder.
O’Neill Rana ARO 11					
*Eulophia dabia* (D. Don) Hoch.	*Amrita Panktikanda* ^NP^, *Hatti Paila* ^NP^, *Mujjatak* ^SN^	Entire Plant	Medicine, Food	[70–73]	As medicine, leaf paste is considered to be a vermicide. The fruit is considered edible.
*Eulophia spectabilis* (Dennst.) Suresh	*Amarkand* ^NP^	Entire Plant	Medicine, Food	[69]	As medicine, leaf paste is considered to be a vermicide. The fruit is considered edible.
*Euphrasia himalayica* Wettst.	*Hare* ^NP^, *Mendosan* ^NP^	Entire Plant	Ritual Object	[29, 73]	Dried bark powder is burned as a ritual incense.
		Inflorescence			
*Helixanthera ligustrina* (Wall.) Danser	*Ainjheru* ^MA^, *Bhringe* ^GU^, *Lisso* ^NP^	Entire Plant	Medicine	Current Study	As medicine, leaf paste is considered to be a vermicide. The fruit is considered edible.
O’Neill Rana ARO 21, 22		Fruit	Food	[29, 62, 65, 68]	
*Helixanthera parasitica* Lour.	*Lisso* ^NP^	Entire Plant	Fodder	Current Study	The entire plant is an alternative fodder.
O’Neill Rana ARO 29				[71, 74, 75]	
*Loranthus lambertianus* Schult.	*Lisso* ^NP^	Entire Plant	Fodder	[71]	The entire plant is an alternative fodder.
O’Neill Rana ARO 30					
*Loranthus odoratus* Wall.	*Ainjeru* ^NP^, *Donglanais* ^TA^, *Khik* ^RA^	Entire Plant	Medicine, Material,	Current Study	Fruit commonly ingested for its laxative and to treat gastric problems. Masticated fruit placed is used in Tamang communities to catch birds, particularly in winter.
O’Neill Rana ARO 5			Food	[26, 62, 75–77]	
*Macrosolen cochinchinensis* (Lour.) Tiegh	*Ainjeru* ^NP^	Entire Plant	Medicine	Current Study	As medicine, leaf paste is consumed to relieve migranes.
O’Neill Rana ARO 7			Fodder	[62, 78]	
*Orobanche aegyptiaca* Pers.	*Nil jhar* ^NP^, *Thokaa* ^TH^, *Thokaraa* ^TH^	Seed	Material	Current Study	Projectile seeds are used as toys in the Terai.
Rana ARO 43				[29]	
*Orobanche alba* Steph. ex Willd.	*Ngoh Droh-shang-tzey* ^TI^, *Juphal* ^NP^	Entire Plant	Medicine	[71]	Pulverized root tissue is applied to burns and scalding wounds and the whole plant is used to relieve vertebrae, waist, and/or leg pain, increase appetite, and heighten the senses.
		Root			
*Osyris quadripartita* Salzm. ex Decne.	*Nundhiki* ^NP^	Leaf	Medicine	[58, 68]	Leaf poultice is used to reduce inflammation, and is also valued as a powerful emetic.
*Osyris wightiana* Wall. ex Wight	*Bakhre Kursani* ^NP^, *Huri* ^NP^, *Jhyalala* ^TA^,	Aerial Parts	Medicine	[29, 79–81]	Whole plant paste is used to reduce inflammation and sprains. Pulverized bark is used to treat indigestion, young, dried leaves can be consumed as tea substitute.
	*Nundhiki* ^NP^, *Nundhikya* ^NP^, *Reskap Sang* ^KH^		Food		
*Pedicularis bifida* (Buch.-Ham. ex D. Don) Pennell	*Pennell* ^NW^	Root	Medicine	[90]	Pulverized root tissue is used to relieve joint pain.
*Pedicularis bicornuta* Klotzsch.	*Lukhru Karpo* ^TI^	Inflorescence	Medicine	[80]	Inflorescence paste is used to treat vaginal and seminal discharges.
*Pedicularis gracilis* Wall. ex Benth subsp. *gracilis*	*Pennell* ^NW^	Root	Medicine	[90]	Pulverized root tissue is used to relieve joint pain.
*Pedicularis oederi* Vahl.	*Phul* ^NP^	Entire Plant	Fodder	[82]	The entire plant is an alternative fodder.
*Pedicularis oliveriana* Prain.	*Lukhru Mhookpo* ^TI^	Inflorescence	Medicine	[82]	Inflorescence used to reduce inflammation, ease gastric pain or disorders, and treat poisoning.
*Pedicularis longiflora* Rudolph subsp. tubiformis	*Lugro Shero* ^GU^, *Sersenlugdu* ^NP^, *Sung* ^NP^	Entire Plant	Medicine	[29, 83, 84]	Entire plant is consumed to treat cough, sore throats, hepatitis, and lymphatic disorders. It is less commonly employed to treat poisioning, seminal/vaginal discharges, and disorders associated with alcoholism.
(Klotzsch) Tsoong	*Lugru Serpo* ^TI^				
*Pedicularis punctata* Decne.	*Lukhru Mugpo* ^TI^, *Mishran* ^SN^	Inflorescence	Medicine	[82]	Tibetan communities use inflorescence to treat fever, cancers, and premature graying of hair.
*Pedicularis pyramidata* Pall. ex. Steven	*L’ang Nah* ^TI^	Entire Plant	Medicine	[82]	Entire plant is consumed to combat fluid retention, including inflammation of bone and the accumulation of serous fluids.
*Pedicularis rhinanthoides* Schrenk	*Phul* ^NP^	Entire Plant	Medicine	[82]	Entire plant is consumed to treat cough, sore throat, hepatitis, and lymphatic disorders. It is less commonly employed to treat poisioning.
*Pedicularis siphonantha* D. Don	*Cheelmootee Swa* ^LI^, *Muferdudolu* ^LI^, *Ponki* ^LI^	Entire Plant	Medicine	[29, 81, 84]	Entire plant is consumed to treat cough, sore throat, hepatitis, and lymphatic disorders. It is less commonly employed to treat poisioning.
	*Lugru Marpo* ^TI^, *Lugru Mugpo* ^TI^				
*Santalum album* L.	*Chandan* ^NP^, *Sirkhandaa* ^NP^, *Sonme Sang* ^KH^	Aerial Parts	Medicine	[67, 70, 82]	Pulverized root tissue is applied to burns and scalding wounds. Tibetan communities use this plant to treat inflammation of lungs, heart, and muscle tissues. Wood oil is particularly important for treating inflammation.
	*Tzenthen Karpo* ^TI^		Fodder		
*Scurrula elata* (Edgeworth) Danser	*Aaingero* ^NP^, *Aainjeru* ^NP^, *Aijeru* ^NP^, *Aijhery* ^LI^, *Ainjer* ^NP^,	Entire Plant	Medicine, Fodder,	Current Study	Entire plant is ground into a paste and used to relieve joint pain and hasten fracture recovery. Fruit is considered to be edible, and is also used for trapping birds.
O’Neill Rana ARO 23, 27, 37	*Bhringe* ^GU^, *Che* ^SA^, *Khik* ^KH^, *Lissau* ^NP^, *Lisso* ^NP^, *Naie* ^TA^	Fruit	Material	[46, 51, 71, 91]	
*Scurrula parasitica* L.	*Ainjeru* ^NP^, *Lisso* ^NP^	Entire Plant	Fodder	Current Study	Fruit is considered edible, and the entire plant is used as fodder. However, some reports detail that shoots induce vomitting and loss of appetite in livestock.
O’Neill Rana ARO 6, 24, 35		Fruit	Food	[26, 62, 68]	
*Scurrula pulverulenta* (Wall.) G. Don	*Ainjeru* ^NE/NP^, *Bhringe* ^GU^	Entire Plant	Medicine	Current Study	Pulverized bark is boiled in water and consumed to treat heptatic disease. Fruit is considered edible, and is also used for trapping birds.
O’Neill Rana ARO 33		Fruit	Fodder	[62]	
*Striga asiatica* (L.) Kuntze	*Cange* ^NP^	Entire Plant	Fodder	[29]	Young stems are considered edible.
		Fruit	Food		
*Taxillus vestitus* (Wall.) Danser	*Ainjeru* ^NP^, *Lisso* ^NP^	Entire Plant	Medicine	Current Study	Plant poultice is used to reduce joint swelling and muscle inflammation.
O’Neill Rana ARO 25			Food	[62, 80]	
*Taxillus umbellifer* (Schult.) Danser	*Ainjeru* ^NP^, *Lisso* ^NP^	Entire Plant	Fodder	Current Study	Fruit is considered edible, and entire plant is sometimes used as fodder. However, shoots are believed to induce vomitting and loss of appetite in livestock.
O’Neill Rana ARO 26		Fruit	Food	[71]	
*Viscum album* L.	*Ainjeru* ^NP/CH^, *Bang* ^TH^, *Hadchud* ^NP^, *Hadjor* ^TH^, *Harchu* ^NW^,	Entire Plant	Medicine	Current Study	*Viscum* species, commonly when bearing fruit, are used to treat a variety of musclo-skeletal disorders and affilctions, including fractured or dislocated fingers, limbs, spines. These treatments are considered most effective when plant is combined with Cinnamon bark and leaves (*Cinnamomum verum*), Nettle root (*Girardinia diversifolia*),*Pinus roxburghii* leaves, and ground bear bones. These substances are mixed and ground into a paste, and are applied to said appendage for a minimum of two months. Leaves are also considered useful for treating earaches, spleen disorders, tetanus, epilepsy, and blood diseases. As medicine, the fruit from both plants is considered to be a favorable laxative, aprhodisiac, and cardiotonic. Ripe fruit were also used as a glue when mixed with water, and is particularly effective for trapping parrots. Ethnoveterinary uses include treatment for cattle wounds and bloating. *Viscum articulatum* is perceived to confer greater medicinal action than *V. album*.
O’Neill Rana ARO 20	*Harchul* ^TA^, *Harchur* ^NP^, *Hajoda* ^NE/NP^, *Harjor* ^GU^,	Fruit	Material	[42, 51, 85, 86]	
	*Gandhamadini* ^SN^, *Jiwantika* ^SN^, *Lisso* ^NP^, *Mecho* ^TA^,		Fodder		
	*Nai* ^TA^,*Sano Hatchur* ^NP^		Food		
*Viscum articulatum* var. *articulatum* Burm.	*Ainjeru* ^NP^, *Hadachu*r^NP^, *Hadjod* ^NP^, *Harchu* ^NW^,	Entire Plant	Medicine, Material,	Current Study	
O’Neill Rana ARO 16, 17	*Bojha* ^RA^, *Gandhmadini* ^SN^, *Kathkomunjga* ^SA^,		Food, Fodder	[47, 69, 71]	
	*Lisso* ^NP^				
*Viscum articulatum* var. *liquidambaricolum* Burm. F.	*Ainjeru* ^NP^, *Hadachur* ^NP^, *Hadjod* ^NP^, *Harchu* ^NW^,	Entire Plant	Medicine, Material,	Current Study	
O’Neill Rana ARO 3	*Bojha* ^RA^, *Gandhmadini* ^SN^, *Kathkomunjga* ^SA^, *Lisso* ^NP^		Food, Fodder	[42]	

^a^Voucher specimen are deposited at TUCH

^b^ CH, Chepang; GU, Gurung; KH, Khaling; MA, Magar; MO, Moosahar; NP, Nepali; NW, Newar; RA, Rai; SA, Satar; SN, Sanskrit; TA, Tamang; TI, Tibetan; TH, Tharu

As medicine, all species were harvested from wild populations and used immediately as fresh material. The entire plant was typically ground to prepare or activate the medicinal potential of each PMP. However, in some cases, dried plant material was also pulverized into a powder (e.g. *Santalum album*). PMP medicinal formulations generally involved single species, and were orally administered as soup (*jhol*) or juice with treated water (*saphaa paani*) or oil (*tel*). A notable exception came from PMPs used to treat fracture and serious hepatic diseases. In these cases, plant paste was directly applied to the site of injury or infection along with other situation-dependent supplements (refer to ‘Notes’ in Table 4). Measurements were not made using a standardized method. Often, highly toxic plants, particularly species in the Balanophoraceae used as vermicide, were dosed based on weight or bodily constitution. Other PMPs were prescribed according to patient preference or tolerability, as many PMP-based herbal medicines are bitter in taste.

Interviews between age groups revealed that the ethnobotanical knowledge surrounding many medicinal PMPs is threatened. All specialist users surveyed in this study were male, and only men in surveyed regions had the opportunity to study under traditional healers. However, this tradition is beginning to change in some Tibetan communities, where women are increasingly encouraged to study under male *amchis*. Overall, the age structure and system of knowledge transmission in many village communities does not promote the promulgation of indigenous knowledge systems in younger generations. All informants under the age of 30 sought the help of allopathic health posts well before traditional medical practitioners, and they only visited traditional healers under extraordinary circumstances. This being said, each our informants under the age of 30 had visited traditional healers as children and were aware of the treatment potential of medicinal plants (*jaributi*). Based on our findings, traditional knowledge is a system evolving within Nepal’s rapidly changing socio-ecological climate, and faces many threats as the state continues to modernize.

### Ethnoecological perceptions of parasitic and mycoheterotrophic plants

Growth habit was most critical factor considered when surveyed groups classified PMPs. For example, Gurung-identifying specialist users collectively classified species in the Balanophoraceae as *prumai*, meaning ‘mushroom-like plants that emerge from the earth’ (Fig. 2, Panels d-f). *Prumai* is not exclusive to PMPs, and it confers a medicinal connotation (*jaributi*) for other organisms such as fungi, but not *Yarsagumba* (*Ophiocordycepssinensis*.). To elaborate, *prumai* grow near or parasitize trees in sacred landscapes, such as holy forests or sacred groves, and have thus become associated with spirits and regional cosmologies. Only specialist users, particularly Gurung *kabres*, made this distinction. Based on our fieldwork, we conclude that this is primarily due to the fact that *prumai* uses are considered arcane. As a result, IBK surrounding these plants remains isolated within specialist circles that do not consistently transmit this knowledge to younger generations. Other more common names, such as the Nepali term *Ek Li ber*, or ‘the one that stands alone’ in old-growth forests, further confirms the importance of growth habit for the identification and use of species in the Balanophoraceae.

Moreover, growth habit is the only factor considered when classifying or distinguishing between *Cuscuta* species. *Cuscuta* are collectively referred to as *Aakash beli* or *Pahilo Lahara*, which translate as ‘sky net’ or ‘yellow climber,’ respectively. Because it lacks leaves and exhibits a vine-like growth habit, this genus does not fit into local ethonotaxonomic schemes. It stands alone as its own plant category simply because it has seeds, and is perceived more generally as a rootless, plant-like mass that forms on the top of shrubs and trees (e.g. Fig. 2, Panel i). As medicine, its vein-like tendrils are complemented by its color and bitter taste to cue its medicinal use for hepatic diseases. Just as hepatic diseases consume the body, turning it yellow and often associated with bitter bile, *Cuscuta* growth habit, as complemented by color and taste, have in many ways become symbolic in medicine for treating similar syndromes.

Finally, parasitic habit is the primary feature used to identify many mistletoe species. Mistletoes are collectively termed *Ainjeru*, meaning ‘scourge’ or plant that debilitates. Although generally isolated to female informants, a variety of cultural beliefs surround this plant and are associated with this name. For instance, several women indicated that burning mistletoe branches leads to goiter, wart-like symptoms, eye problems, and family debt. If brought into one’s house, mistletoes could also lead to hauntings. All symptoms appear to be correlated with the perceived biology of the plant, or the bulbous masses and wart-like protrusions that *Ainjeru* inflicts upon its host. An exception to this rule exists for Phulchoki-area Tamangs, who believe mistletoe-infected wood confers good luck during gambling [62]. Women were the primary user groups for mistletoes, as they were responsible for collecting fodder plants for buffalo and cows.

Plant utility is a secondary identifier for mistletoe species in the Viscaceae, especially *Viscum album* and *V. articulatum*, and such knowledge remains isolated to male user groups. *Harchor*, a Nepali term meaning ‘bone binder’ or a substance that facilitates the repair of bones, denotes these plants’ common use for treating fracture. When describing *Harchor*’s medicinal use, male informants consistently referenced the plants growth habit along with its potency. Because they create bulbous masses on tree branches, making a thinner branch thicker, they have been appropriated into medicine for treating fracture. Together with the joint-like nature of *V. articulatum* leaves, growth habit appears to be the primary feature signaling Viscaceae use. Beyond growth habit, leaf shape, preferred host plant, and flower are used to further distinguish mistletoe species based on alternative utilities.

**Fig. 4:**
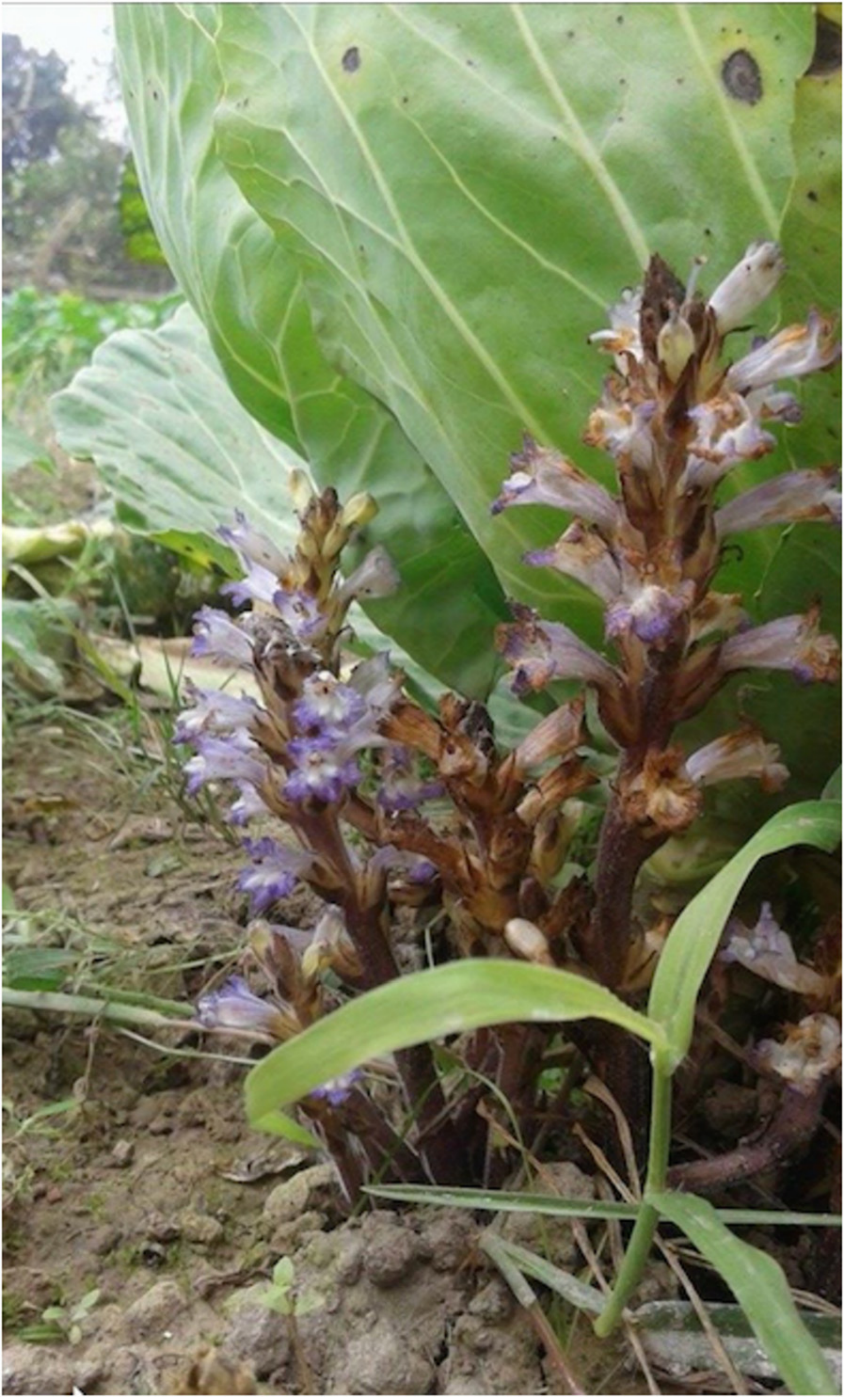
*Orobanche aegyptiaca*, or *bandaarphul* (monkey flower), parasitizing *Brassica oleracea* in the Western Terai

In the Terai, *Orobanche* and *Striga* species were generally known as variations of the Nepali word *jhar*, meaning ‘grass’ or ‘grass-like weedy plant’. This lack of differentiation mirrors these plants’ limited IBK, including the species we surveyed: *Orobanche aegyptiaca* (Fig. 4), *Striga gesneroides*, and *S. asiatica. Orobanche* spp. in some areas of the central Terai are known as *Thokara* or *Thoka*, meaning swollen rhizome. This again describes these plants’ parasitic organ know as *haustoria*, and, inherently, its growth habit. In other regions, *Orobanche* spp. are referred to as *Bandaar Phul*, or monkey flower, due to its projectile fruit and dispersion method.

### Parasitic plants and agricultural development

Based on fieldwork with Plant Quarantine Officers, species in the Orobanchaceae pose a potential threat to agricultural production in Nepal’s Terai region. In particular, *Orobanche* and *Striga* spp. were widely recognized as invasive weeds infecting many crop systems, including cabbage, maize, millet, potato, and sugarcane plots (Fig. 4). Qualitatively, their populations were reported to have increased in recent decades, with more frequent and widespread ranges today than in previous decades. Our informants primarily cited that increased host densities (i.e. spread of agriculture) facilitated this spread. Similar reports were received in Kaski’s Community Forest systems regarding mistletoe species (Loranthaceae). For conservation purposes, future studies should focus on parasite infestation and the expansion of agricultural development in Nepal, including changes in forest habitat, fallow lands, and wetland ecosystems. Exacerbated by climate change, PMP are likely to have range-shifts into higher altitude fields, threatening native biodiversity and the integrity of historical ecosystems.

Increased PMP population sizes and densities are reported throughout Nepal. Both community foresters and forest users groups noted marked increases in Loranthaceae populations on *Alnus nepalensis*, *Prunus cornuta*, *Pyrularia edulis*, *Symplocos ramosissima*, *Berberis* spp., and *Quercus* spp. This appears due to the fact that older trees are less frequently felled, and these trees are most likely to become parasitized [87, 88]. Community forestry programs have thus ironically preserved Nepalese forests, however has in turn facilitated increased parasitism. An exception to PMP population increases were seen in declined *Cuscuta* populations, as well as its use as medicine, due to habitat loss and over-exploitation of *Cuscuta* host plants for fodder or fuel.

## Conclusions

Ethnobotanical analyses provide insight into how indigenous groups manage and perceive natural resources based on traditional relationships to the environment. They can provide crucial details on the population ecology and economic importance of many species, and are thus crucial when developing environmental management programs in regions such as the central Himalaya. Our study revealed that many Nepalese people possessed a great deal of IBK on PMPs, in spite of the fact that many PMPs are not longer used a medicine. Our study also depicts the heterogeneity of IBK in Nepal as stratified within and among ethnic groups and age cohorts. Both species diversity and the traditional knowledge that surrounds them are important factors to consider when designing future conservation projects.

## Additional files

Additional file 1:
**Parasitic plant species found in Nepal.** Nepal specific data, including host species are presented. (PDF 128 kb) Additional file 2:
**Mycoheterotrophic plant species found in Nepal.** Nepal specific data, including host species are presented. (PDF 52 kb) Additional file 3:
**Plant distribution maps of parasitic and mycoheterotropic plant species found in Nepal.** (PDF 106616 kb) Additional file 4:
**Semi-structured questionnaire for an ethnobotanical analysis of parasitic plants in the Nepal Himalaya.** (PDF 154 kb) Additional file 5:
**Voucher records of collected parasitic plant species from Central and Eastern Nepal.** (PDF 54 kb)
