# Supplementary material for: An ethnobotanical analysis of parasitic plants (Parijibi) in the Nepal Himalaya
Source: J Ethnobiol Ethnomed. 2016 Feb 24;12:14. doi: 10.1186/s13002-016-0086-y (PMC4765049; doi:10.1186/s13002-016-0086-y)
Supplement: Additional file 1: — Parasitic plant species found in Nepal. Nepal specific data, including host species are presented. (PDF 128 kb) [file 13002_2016_86_MOESM1_ESM.pdf]

Appendix I. Parasitic plant species found in Nepal. Nepal-specific data, including host species, are presented.
(Shrestra 1998; Oleg and Stainton 2000; Manandhar 2002; Press et al. 2000), KATH 2013;
TUCH 2013; TPL 2013; eFloras 2013; Tropicos 2013).

Key: Distribution information is abbreviated by E (Eastern), C (Central), and W (West)
based on Nepal's three vegetative zones. N/A denotes where information is not available.

SNo.	Species	Family	Distribution	Altitude (m)	Habit	Recorded Hosts	Flowering Time	Fruiting Time
1	***Dufrenoya granulata*** (Hook. F. & Thomson) Stauffer	Amphorogynaceae	C.E.	1600 - 2400	Facultative root hemiparasite	Fagaceae, Malvaceae, Moraceae	May - August	September - October
2	***Dufrenoya platyphylla*** (Spreng.) Stauffer.	Amphorogynaceae	C.E.	1200 - 2000	Facultative root hemiparasite	Fagaceae, Malvaceae, Moraceae	May - August	September - October
3	***Balanophora dioica*** R. Brown ex Royle	Balanophoraceae	C.E.	400 - 2600	Root holoparasite	Ericaceae *(Rhododendron* spp)	August - October	October - November
4	***Balanophora involucrata*** J.D. Hooker	Balanophoraceae	W.C.E.	2900 - 3400	Root holoparasite	Ericaceae *(Rhododendron* spp)	July - August	August - September
5	***Balanophora polyandra*** Griffith.	Balanophoraceae	E.	1400 - 3000	Root holoparasite	Ericaceae *(Rhododendron* spp)	August - October	September - October
6	***Rhopalocnemis phalloides*** Jungh.	Balanophoraceae	E.	2100 - 2200	Root holoparasite	Araliaceae (*Dendropanax spp.*), Euphorbiaceae, *Pyrularia edulis*, *Symplocos ramosissima*, *Quercus* spp.	N/A	N/A
7	***Pyrularia edulis*** (Wallich) A. Candolle	Cervantesiaceae	W.C.E.	1600 -1800	Facultative root hemiparasite	Fagaceae, Moraceae, Santalaceae (*Acanthosyris*, *Pyrularia*, *Scleropyrum*)	December - April	August - November
8	***Cuscuta chinensis*** Lam.	Convolvulaceae	C.	1300	Stem holoparasite	Amaranthaceae (*Alternanthera philoxeroides*), Asteraceae, Berberidaceae (*Berberis* spp.), Chenopodiaceae, Euphorbiaceae (*Jatropha curacas*), Hydrangeaceae (Dichroa febrifuga), Lamiaceae (*Callicarpa macrophylla)*, Myrsinaceae (*Maesa chisia)*, Rutaceae (*Citrus* spp.)	December - April	August - November
9	***Cuscuta europaea*** var. ***indica*** Engelm.	Convolvulaceae	W.C.	2400 - 4000	Stem holoparasite	Asteraceae (*Artemesia indica*), Berberidaceae (Berberis spp.), Dipsacaceae (Dipsacus inermis), Fagaceae (*Quercus semicarpifolia*), Malvaceae, Pinaceae (*Pinus wallichaina*), Rutaceae (*Zanthoxylem* spp.), Salicaceae (*Salix* spp.)	September - October	September - October
10	***Cuscuta europaea*** var. ***nepalensis*** Yunker.	Convolvulaceae	W.	2300	Stem holoparasite	Range of plant families including Malvaceae	September - October	September - October
11	***Cuscuta gigantea*** Griffith.	Convolvulaceae	W.	2550	Stem holoparasite	Range of plant families including Malvaceae, Tamaricaceae (*Tamarix* spp)	September - October	September - October
12	***Cuscuta reflexa*** var. ***brachystigma*** Englem.	Convolvulaceae	C.E.	200 - 2200	Stem holoparasite	Range of plant families including Malvaceae, Tamaricaceae (*Tamarix* spp)	September - October	September - October
13	***Cuscuta reflexa*** var. ***reflexa*** Roxb.	Convolvulaceae	W.C.E.	1100 - 3100	Stem holoparasite	Range of plant families including Balsaminaceae *(Impatiens* spp.,), Berberaceae (*Berberis* spp.), Lamiaceae (*Vitex negundo*), Malvaceae, Tamaricaceae (*Tamarix* spp.)	September - October	September - October
14	***Arceuthobium minutissimum*** Hook.f.	Loranthaceae	W.	3000 - 4000	Obligate stem hemiparasite	Pinaceae (*Pinus gerardiana*)		
15	***Dendrophthoe falcata*** (L.f.) Etting.	Loranthaceae	W.C.E.	150 - 900	Obligate stem hemiparasite	Anacardiaceae (*Mangifera indica*), Euphorbiaceae Malvaceae (Bombax spp.), Moraceae (*Ficus* spp.)	June - December	June - December
16	***Dendrophthoe pentandra*** (L.) Miq	Loranthaceae	E.	1200	Obligate stem hemiparasite	Anacardiaceae, Euphorbiaceae, Malvaceae (*Bombax* spp.) Moraceae, and Rutaceae, Theaceae (*Schimia wallichii)*	June - December	June - December
17	***Helixanthera ligustrina*** (Wall.) Danser	Loranthaceae	W.C.	900 - 1700	Obligate stem hemiparasite	Euphorbiaceae (*Macaranga* spp.; *Mallotus phillipennsis*), Meliaceae (Melia azedarach), Rutaceae (*Zanthoxylum* spp.)	N/A	N/A
18	***Helixanthera parasitica*** Lour.	Loranthaceae	E.	900 - 1200	Obligate stem hemiparasite	Euphorbiaceae, Fagaceae (*Castanopsis indica*), Lauraceae, Meliaceae, Moraceae,Rosaceae, Rutaceae, Theaceae	January - July	May - August
19	***Loranthus lambertianus*** J.H. Schultes	Loranthaceae	W.C.E.	900	Obligate stem hemiparasite	Fagaceae (*Quercus* spp.)	January - April	January - April
20	***Loranthus odoratus*** Wall.	Loranthaceae	C.E.	1600 - 2400	Obligate stem hemiparasite	Fagaceae *(Quercus, Castanopsis*)	February - March	July
21	***Macrosolen cochinchinensis*** (Lour.) Tiegh	Loranthaceae	W.E.	250 - 1700	Obligate stem hemiparasite	Moraceae, Euphorbiaceae, Fagaceae, Lauraceae Meliaceae, Rosaceae, Rutaceae, Theaceae	February - June	May - August
22	***Scurrula cordifolia*** (Wall) G. Don	Loranthaceae	W.C.E	1500 - 2200	Obligate stem hemiparasite	Apocynaceae, Euphorbiaceae, Fabaceae, Fagaceae, Lythraceae, Moraceae, Punicaceae, Rosaceae, Rutaceae, Sapindaceae, Theaceae, and Ulmaceae	N/A	N/A
23	**Scurrula elata** (Edgeworth) Danser	Loranthaceae	W.C.E.	1600 - 2700	Obligate stem hemiparasite	Anacardiaceae (*Mangifera indica; Buchanania latifolia*),	May - July	July - August

						Aquifoliaceae (*Ilex* spp.), Ericaceae (*Gaultheria* spp., *Rhododendron* spp.), Fagaceae (*Quercus* spp.), Rosaceae (*Prunus* spp.), Vibernaceae		
24	***Scurrula parasitica*** L.	Loranthaceae	W.C.E.	200 - 2200	Obligate stem hemiparasite	Apocynaceae, Euphorbiaceae, Ericaceae (*Rhododendron* spp., *Symplocos* spp.) Fabaceae, Fagaceae, Lamiaceae (*Vitex negundo*), Lythraceae, Moraceae, Punicaceae, Rosaceae, Rutaceae, Sapindaceae, Theaceae, and Ulmaceae	January - October	January - October
25	***Scurrula parasitica*** var. ***graciliflora*** (Roxb. Ex Schult.) H.S. Kiu	Loranthaceae	W.C.E.	2000 - 2200	Obligate stem hemiparasite	Apocynaceae, Euphorbiaceae, Fabaceae, Fagaceae, Lythraceae, Moraceae, Punicaceae, Rosaceae, Rutaceae, Sapindaceae, Theaceae, Ulmaceae	N/A	N/A
26	***Scurrula pulverulenta*** (Wall.) G. Don	Loranthaceae	W.C.E.	200 - 1400	Obligate stem hemiparasite	Lauraceae, Euphorbiaceae, Rutaceae	March - August	August - March
27	***Taxillus umbellifer*** (Schult.) G.Don	Loranthaceae	E.	1500 - 2100	Obligate stem hemiparasite	Lauraceae, Euphorbiaceae, Rutaceae	August	September
28	**Taxillus vestitus** (Wall.) Danser	Loranthaceae	W.E.	150 - 1500	Obligate stem hemiparasite	Fagaceae	July - November	July - November
29	**Olax nana** Wall ex Benth	Olacaceae	C.	1900	Facultative root hemiparasite	N/A	April - May	April - May
30	**Erythropalum vagum** (Griff.) Mast	Olacaceae	C.	3000 - 4000	Facultative root hemiparasite	N/A	N/A	N/A
31	**Cansjera rheedii** J.F. Gmel.	Opiliaceae	C.	600	Obligate root hemiparasite	N/A	N/A	N/A
32	***Lepionurus sylvestris*** Blume.	Opiliaceae	E.	300	Facultative root hemiparasite	N/A	July - November	July - November
33	***Aeginetia indica*** L.	Orobanchaceae	W.C.E	250 - 1700	Root holoparasite	Poaceae (*Miscanthus*, *Saccharum*)	April - August	August - October.
34	***Aeginetia pedunculata*** (Roxb.) Wall.	Orobanchaceae	C,	250	Root holoparasite			
35	***Boschniakia himalaica*** Hook. & Thomson ex Hook.	Orobanchaceae	C.E.	2900 - 4300	Root holoparasite	Ericaceae (Rhododendron *spp.*)	April - June	June - September
36	***Buchnera cruciata*** Hamilt.	Orobanchaceae	C.	1400 - 1600	Root holoparasite	N/A	January - April	Jamuary - April
37	***Buchnera hispida*** Buch.-Ham ex D. Don	Oronachnaceae	W.C.E	1500 - 1900	Root holoparasite	N/A	N/A	N/A
38	***Centranthera grandiflora*** Benth.	Orobanchaceae	E.	200 1800	Faculatative root hemiparasite	N/A	July - September	September
39	***Centranthera cochinchinensis var. nepalensis (D. Don) Merr.***	Orobanchaceae	W.C.E	200 - 1800	Faculatative root hemiparasite	N/A	August - October.	August - October.
40	***Euphrasia himalayica*** Wettst.	Orobanchaceae	W.C.E.	3200 - 4200	Facultative root hemiparasite	N/A	June	June
41	***Euphrasia jaeschkei*** Wettst.	Orobanchaceae	W.C.	3200 - 3400	Facultative root hemiparasite	N/A	June	June
42	***Euphrasia multiflora*** Pennell	Orobanchaceae	C.	3200	Facultative root hemiparasite	N/A	June	June
43	***Euphrasia nepalensis*** Pugsley.	Orobanchaceae	W.C.	2800 - 4300	Facultative root hemiparasite	N/A	June	June
44	***Euphrasia platyphylla*** Pennell	Orobanchaceae	W. C. E	2700- 3700	Facultative root hemiparasite	N/A	June	June
45	***Euphrasia schlagintweitii*** Wettst	Orobanchaceae	W.C.E	3000 - 3600	Facultative root hemiparasite	N/A	June	June
46	***Euphrasia simplex*** D.Don	Orobanchaceae	C.	2500 - 2900	Facultative root hemiparasite	N/A	June	June
47	***Leptorhabdos parviflora*** (Benth.) Benth.	Orobanchaceae	W.C.	1700 - 2300	Faculatative root hemiparasite	N/A	July - August	July - August
48	***Orobanche aegyptiaca*** Pers	Orobanchaceae	W.C.	150 - 3200	Root holoparasite	Cannabaceae (*Cannabis sativa*), Cucurbitaceae (*Cucumis melo*)	April - June	June - August
49	***Orobanche alba*** Steph. Ex Willd.	Orobanchaceae	W.C.	2300 - 3700	Root holoparasite	Brassicaceae	April - June	July - August
50	***Orobanche cernua var. cernua*** Loefl.	Orobanchaceae	C.	2400 - 2900	Root holoparasite	N/A	N/A	N/A
51	***Orobanche cernua var. nepalensis*** Loefl.	Orobanchaceae	C.	2400 - 2900	Root holoparasite	N/A	N/A	N/A
52	***Orobanche coerulescens*** Steph.	Orobanchaceae	W.C.	2100 - 2700	Root holoparasite	Asteraceae (*Artemesia* spp.)	N/A	N/A
53	***Orobanche ramosa*** L.	Orobanchaceae	W.	Reported but never collected.	Root holoparasite	N/A	N/A	N/A
54	***Orobanche solmsii*** C. B. Clarke ex Hook. f	Orobanchaceae	W.C.	3400	Root holoparasite	N/A	May - June	June - August
55	***Pedicularis alaschanica*** Maxim.	Orobanchaceae	C.	3900 - 5100	Faculatative root hemiparasite	N/A	June - August	September
56	***Pedicularis alaschanica*** subsp. ***tibetca*** (Maxim.) Tsoong	Orobanchaceae	C.	4200 - 4800	Faculatative root hemiparasite	N/A	N/A	N/A
57	***Pedicularis albiflora*** (Hook f.) Prain	Orobanchaceae	E.	4000 - 4500	Faculatative root hemiparasite	N/A	N/A	N/A

58	***Pedicularis annapurnensis*** T.Yamaz	Orobanchaceae	C.	4200	Faculatative root hemiparasite	N/A	N/A	N/A
59	***Pedicularis anserantha*** T.Yamaz var. ***anserantha***	Orobanchaceae	W.C.	3730 - 4000	Faculatative root hemiparasite	N/A	N/A	N/A
60	***Pedicularis anserantha*** T.Yamaz var. ***elevantogaleata***	Orobanchaceae	W.C.	3800	Faculatative root hemiparasite	N/A	N/A	N/A
61	***Pedicularis bicornuta*** Klotzsch	Orobanchaceae	C.E.	5300	Faculatative root hemiparasite	N/A	N/A	N/A
62	***Pedicularis bifida*** (Buch.-Ham.ex D. Don) Pennell	Orobanchaceae	W.C.E.	1600 - 2550	Faculatative root hemiparasite	N/A	N/A	N/A
63	***Pedicularis brevifolia*** D. Don	Orobanchaceae	C.E.	2510 - 4300	Faculatative root hemiparasite	N/A	N/A	N/A
64	***Pedicularis breviscaposa*** T. Yamaz	Orobanchaceae	C.	3000 - 4000	Faculatative root hemiparasite	N/A	N/A	N/A
65	***Pedicularis chamissonoides*** T. Yamaz	Orobanchaceae	C.	3800	Faculatative root hemiparasite	N/A	N/A	N/A
66	***Pedicularis cheilanthifolia*** Schrenk.	Orobanchaceae	W.	2100 - 5200	Faculatative root hemiparasite	N/A	June - August	July - September
67	***Pedicularis cheilanthifolia*** subsp. ***Nepalensis*** T. Yamaz	Orobanchaceae	W.	4200 - 4900	Faculatative root hemiparasite	N/A	N/A	N/A
68	***Pedicularis clarkei*** Hook.	Orobanchaceae	C.E.	3600 - 4200	Faculatative root hemiparasite	N/A	N/A	N/A
69	***Pedicularis collata*** Prain.	Orobanchaceae	E.	4200 - 4900	Faculatative root hemiparasite	N/A	N/A	N/A
70	***Pedicularis confertiflora*** Prain.	Orobanchaceae	W.C.E.	4100 - 4900	Faculatative root hemiparasite	N/A	July - September	August - October
71	***Pedicularis cornigera*** T. Yamaz	Orobanchaceae	E.	4100 - 4700	Faculatative root hemiparasite	N/A	N/A	N/A
72	***Pedicularis denudata*** Hook f.	Orobanchaceae	C.	3870 - 4300	Faculatative root hemiparasite	N/A	N/A	N/A
73	***Pedicularis diffusa*** Prain	Orobanchaceae	C.	3300 - 4960	Faculatative root hemiparasite	N/A	May - July	May - July
74	***Pedicularis elwesii*** Hook f.	Orobanchaceae	C.E.	3000 - 4800	Faculatative root hemiparasite	N/A	June - August	August - September
75	***Pedicularis excelsa*** Hook f.	Orobanchaceae	C.	3400	Faculatative root hemiparasite	N/A	August	August - September
76	***Pedicularis flexuosa*** Hook f.	Orobanchaceae	C.E.	3000 - 4100	Faculatative root hemiparasite	N/A	June - August	July - September
77	***Pedicularis furfuracea*** Wall.ex Benth.	Orobanchaceae	C.E.	2700 - 4100	Faculatative root hemiparasite	N/A	June - July	July - August
78	***Pedicularis gibbera*** Prain.	Orobanchaceae	E.	3600 - 3700	Faculatative root hemiparasite	N/A	N/A	N/A
79	***Pedicularis globifera*** Hook f.	Orobanchaceae	W.C.	3900 - 5500	Faculatative root hemiparasite	Poaceae (*Kobresia* spp.)	June - October	June - October
80	***Pedicularis gracilis*** Wall ex Benth subsp. ***gracilis***	Orobanchaceae	W.	2100 - 3300	Faculatative root hemiparasite	N/A	N/A	N/A
81	***Pedicularis gracilis*** Wall ex Benth subsp. ***macrocarpa*** (Prain) Tsoong	Orobanchaceae	C	3320	Faculatative root hemiparasite	N/A	N/A	N/A
82	***Pedicularis gracilis*** subsp. ***stricta*** Wall. Ex Benth	Orobanchaceae	W.	2300 - 2700	Faculatative root hemiparasite	N/A	N/A	N/A
83	***Pedicularis gruiflora*** T. Yamaz	Orobanchaceae		4700	Faculatative root hemiparasite	N/A	N/A	N/A
84	***Pedicularis heydei*** Prain	Orobanchaceae	W.C.	4000 - 5000	Faculatative root hemiparasite	N/A	N/A	N/A
85	***Pedicularis hoffmeisteri*** Klotzsch.	Orobanchaceae	W.C.E.	2300 - 3900	Faculatative root hemiparasite	N/A	N/A	N/A
86	***Pedicularis hookeriana*** Wall.	Orobanchaceae	W.C.	2500 - 4800	Faculatative root hemiparasite	N/A	N/A	N/A
87	***Pedicularis instar*** Prain ex Maxim	Orobanchaceae	E.	2600 - 4200	Faculatative root hemiparasite	N/A	N/A	N/A
88	***Pedicularis integrifolia*** Hook. F.	Orobanchaceae	C.	4500 - 4800	Faculatative root hemiparasite	N/A	June - July	July - September
89	***Pedicularis kansuensis*** Maxim.	Orobanchaceae	W.E.	3800 - 4100	Faculatative root hemiparasite	N/A	N/A	N/A
90	***Pedicularis klotzschii*** Hurus.	Orobanchaceae	W.C.	2300 - 4500	Faculatative root hemiparasite	N/A	June - August	July - September
91	***Pedicularis koshiensis*** T. Yamaz	Orobanchaceae	W. C.	2500 - 4800	Faculatative root hemiparasite	N/A	N/A	N/A
92	***Pedicularis lachnoglossa*** Hook. F.	Orobanchaceae	E.	4000 - 4500	Faculatative root hemiparasite	N/A	June - July	August
93	***Pedicularis longiflora*** Rudolph subsp. ***tubiformis*** (Klotzsch) Tsoong	Orobanchaceae	W.C.E.	3300 - 5000	Faculatative root hemiparasite	N/A	May - October	May - October
94	***Pedicularis megalantha*** D. Don	Orobanchaceae	C.E.	2800 - 4300	Faculatative root hemiparasite	N/A	June - August	July - September
95	***Pedicularis megalochila*** Li subsp. ***longituba***	Orobanchaceae	E.	3500 - 4000	Faculatative root hemiparasite	N/A	N/A	N/A

96	***Pedicularis microcalyx*** Hook f.	Orobanchaceae	C.E.	3700 - 5500	Faculatative root hemiparasite	N/A	June - August	July - August
97	***Pedicularis mollis*** Wall .ex Benth.	Orobanchaceae	W.C.E.	2600 - 4500	Faculatative root hemiparasite	N/A	July - September	July - September
98	***Pedicularis muguensis*** T. Yamaz.	Orobanchaceae	W.	3600 - 4350	Faculatative root hemiparasite	N/A	N/A	N/A
99	***Pedicularis muscoides*** H.L Li	Orobanchaceae	W.C.E.	4500 - 5700	Faculatative root hemiparasite	N/A	June - August	July - September
100	***Pedicularis muscoides*** subsp. ***himalayca*** T. Yamaz.	Orobanchaceae	C.	4800 - 5850	Faculatative root hemiparasite	N/A	N/A	N/A
101	***Pedicularis nana*** C. E. C. Fisch	Orobanchaceae	C.	4800 - 5100	Faculatative root hemiparasite	N/A	N/A	N/A
102	***Pedicularis nepalensis*** Prain.	Orobanchaceae	C.E.	3100 - 4500	Faculatative root hemiparasite	N/A	N/A	N/A
103	***Pedicularis nodosa*** Pennell	Orobanchaceae	C.	3400 - 4300	Faculatative root hemiparasite	N/A	N/A	N/A
104	***Pedicularis odontoloma*** T. Yamaz	Orobanchaceae	W.	4700	Faculatative root hemiparasite	N/A	N/A	N/A
105	***Pedicularis oederi*** subsp. ***oederi*** var. ***heteroglossa*** Vahl.	Orobanchaceae	W.C.E.	3000 - 5500	Faculatative root hemiparasite	N/A	N/A	N/A
106	***Pedicularis ophiocephala*** Maxim.	Orobanchaceae	W.	3500 - 4000	Faculatative root hemiparasite	N/A	N/A	N/A
107	***Pedicularis oxyrhyncha*** T. Yamaz.	Orobanchaceae	C.E.	3900 - 4400	Faculatative root hemiparasite	N/A	N/A	N/A
108	***Pedicularis pantlingii*** subsp. ***pantlingii*** Prain.	Orobanchaceae	C.E.	3800 - 4200	Faculatative root hemiparasite	N/A	July - August	August - September
109	***Pedicularis paradoxa*** (Prain) T. Yamaz	Orobanchaceae	E.	4300 - 4400	Faculatative root hemiparasite	N/A	N/A	N/A
110	***Pedicularis pauciflora*** (Prain) Pennell	Orobanchaceae	E.	3600 - 4600	Faculatative root hemiparasite	N/A	N/A	N/A
111	***Pedicularis pectinata*** Wall.ex Benth.	Orobanchaceae	W.	3000 - 4000	Faculatative root hemiparasite	N/A	N/A	N/A
112	***Pedicularis poluninii*** P.C. Tsoong	Orobanchaceae	C.	4000 - 4700	Faculatative root hemiparasite	N/A	N/A	N/A
113	***Pedicularis porrecta*** Wall.	Orobanchaceae	W.	3000 - 4000	Faculatative root hemiparasite	N/A	N/A	N/A
114	***Pedicularis pseudoregeliana*** P.C. Tsoong	Orobanchaceae	C.E.	4000 - 4600	Faculatative root hemiparasite	N/A	N/A	N/A
115	***Pedicularis regeliana*** Prain.	Orobanchaceae	C.E.	3200 - 4500	Faculatative root hemiparasite	N/A	N/A	N/A
116	***Pedicularis rhinanthoides*** Schrenk subsp. ***labellata*** (Jacquem.) Prain ex Pennell	Orobanchaceae	C.	3000 - 4100	Faculatative root hemiparasite	N/A	N/A	N/A
117	***Pedicularis rhinanthoides*** subsp. ***tibetica*** (Bonati) P.C. Tsoong	Orobanchaceae	E.	4200 - 4600	Faculatative root hemiparasite	N/A	May - July	August - September
118	***Pedicularis roylei*** subsp. ***megalantha*** P.C. Tsoong	Orobanchaceae	W.	3800 - 5600	Faculatative root hemiparasite	N/A	N/A	N/A
119	***Pedicularis roylei*** subsp. ***roylei*** Maxim.	Orobanchaceae	W.C.E	3600 - 4000	Faculatative root hemiparasite	Ercicaceae (*Rhododendron* spp.)	July - August	August - September
120	***Pedicularis roylei*** var. ***speciosa*** (Prain) T. Yamaz	Orobanchaceae	W.C.E.	3600 - 4000	Faculatative root hemiparasite	N/A	N/A	N/A
121	***Pedicularis schizorrhyncha*** Prain.	Orobanchaceae	C.	4200 - 4900	Faculatative root hemiparasite	N/A	August - September	September
122	***Pedicularis scullyana*** Prain ex Maxim.	Orobanchaceae	W.C.E.	3400 - 5000	Faculatative root hemiparasite	N/A	N/A	N/A
123	***Pedicularis sectifolia*** T. Yamaz.	Orobanchaceae	W. C. E.	3400 - 4300	Faculatative root hemiparasite	N/A	N/A	N/A
124	***Pedicularis sikkimensis*** Bonati ex W. W. Sm.	Orobanchaceae	C.	4000 - 4450	Faculatative root hemiparasite	N/A	N/A	N/A
125	***Pedicularis siphonantha*** D. Don	Orobanchaceae	W.C.E.	3000 - 4400	Faculatative root hemiparasite	N/A	June - July	July - August
126	***Pedicularis tamurensis*** T. Yamaz.	Orobanchaceae	E.	2800 - 3400	Faculatative root hemiparasite	N/A	N/A	N/A
127	***Pedicularis tenuicaulis*** Prain	Orobanchaceae	E.	4000	Faculatative root hemiparasite	N/A	July - August	August - September
128	***Pedicularis terrenoflora*** T. Yamaz.	Orobanchaceae	E.	2000 - 2200	Faculatative root hemiparasite	N/A	N/A	N/A
129	***Pedicularis trichodonta*** T. Yamaz.	Orobanchaceae	E.	3600 - 4500	Faculatative root hemiparasite	N/A	N/A	N/A
130	***Pedicularis trichoglossa*** Hook. F.	Orobanchaceae	W.C.E.	4000 - 4500	Faculatative root hemiparasite	N/A	July - August	August - September.
131	***Pedicularis tsoongii*** T. Yamaz	Orobanchaceae	W.	3800	Faculatative root hemiparasite	N/A	N/A	N/A
132	***Pedicularis wallichii*** Bunge	Orobanchaceae	W.C.E.	4000 - 4500	Faculatative root hemiparasite	N/A	June - August	August
133	***Pedicularis yalungensis*** T. Yamaz.	Orobanchaceae	C.	4300 - 5300	Faculatative root hemiparasite	N/A	N/A	N/A
134	***Pedicularis yamazakiana*** R. R. Mill	Orobanchaceae	W.	3405	Facultative root hemiparasite	N/A	N/A	N/A

135	***Phtheirospermum glandulosum*** (Benth.) Benth ex Hook. F.	Orobanchaceae	W.E.	1800 - 2600	Faculatative root hemiparasite	N/A	N/A	N/A
136	***Striga angustifolia*** (D. Don) C. J. Saldanha	Orobanchaceae	W.C.	580 - 1900	Obligate root hemiparasite	N/A	September - December	September - December
137	***Striga asiatica*** (L.) Kuntze	Orobanchaceae	W.C.E.	500 - 1500	Obligate root hemiparasite	Poaceae *(Saccharrum* spp.)	September - December	September - December.
138	***Striga gesnerioides*** (Willd.) Vatke	Orobanchaceae	W.	1350 - 1700	Obligate root hemiparasite	Poaceae *(Saccharrum* spp.)	N/A	N/A
139	***Striga masuria*** (Buch.-Ham.ex Benth.) Benth.	Orobanchaceae	E.	900 - 1100	Obligate root hemiparasite	Poaceae *(Saccharrum* spp.)	N/A	N/A
140	***Osyris quadripartita*** Salzm. ex Decne.	Santalaceae	W.C.	900 - 2200	Facultative root hemiparasite	N/A	April - June	October
141	***Osyris wightiana*** (Santalaceae)	Santalaceae	C.E.	1100 - 2600	Facultative root hemiparasite	N/A	N/A	N/A
142	***Thesium emodi*** Hendrych	Santalaceae	C.	2900 - 4100	Facultative root hemiparasite	N/A	N/A	N/A
143	***Thesium himalense*** Royle	Santalaceae	C.	1300 - 4000	Facultative root hemiparasite	N/A	June	August - September
144	***Santalum album*** L.	Santalaceae	W.C.E.	600 - 1200	Facultative root hemiparasite	N/A	N/A	N/A
145	**Schoepfia fragrans** Wall.	Schoepfiaceae	C.E.	1400 - 1800	Obligate root hemiparasite	N/A	September - December	October - March
146	***Viscum articulatum*** var. ***articulatum*** Burm.	Viscaceae	W.C.	1500 - 2100	Obligate stem hemiparasite	Mauraceae, Lauraceae, Lythraceae (*Punica granatum*), Oleaceae	January - December	January - December
147	***Viscum articulatum*** var. ***liquidambaricolum*** Burm. F.	Viscaceae	W.C.E.	200 - 2200	Obligate stem hemiparasite	Cornaceae (*Cornus capitata*), Fagaceae (*Quercus glauca, Quercus semicarpifolia*), Ericaceae (*Rhododendron* spp.), Lauraceae, Lythraceae, Moraceae (*Ficus religiosa*), Oleaceae, Rosaceae (*Prunus cornuta*)	January - December	January - December
148	***Viscum loranthi*** Elmer	Viscaceae	W.C.	1200 - 2600	Obligate stem hemiparasite	Lauraceae, Loranthaceae (*Macrosolen cochinchinensis*), Lythraceae, Moraceae, Oleaceae,	March - May	March - May
149	***Viscum monoicum*** Roxb.ex DC.	Viscaceae	C.	980 - 1000	Obligate stem hemiparasite	Dipterocarpaceae (*Shorea robusta*), Lauraceae, Lythraceae, Moraceae, Oleaceae,	March - December	March - December
150	***Viscum multinerve*** (Hayata) Hayata	Viscaceae	C.	1800	Obligate stem hemiparasite	Fagaceae (*Castanopsis indica*), Lauraceae, Lythraceae, Moraceae Oleaceae	April - December	April - December
151	***Viscum album*** L.	Viscaceae	W.C.	600 - 2650	Obligate stem hemiparasite	Lauraceae, Lythraceae, Moraceae, Oleaceae, Rosaceae (Pyrus spp.)	November - March	July - December
