# Supplementary material for: An ethnobotanical analysis of parasitic plants (Parijibi) in the Nepal Himalaya
Source: J Ethnobiol Ethnomed. 2016 Feb 24;12:14. doi: 10.1186/s13002-016-0086-y (PMC4765049; doi:10.1186/s13002-016-0086-y)
Supplement: Additional file 2: — Mycoheterotrophic plant species found in Nepal. Nepal specific data, including host species are presented. (PDF 52 kb) [file 13002_2016_86_MOESM2_ESM.pdf]

Appendix II. Parasitic plant species found in Nepal. Nepal-specific data, including host species, are presented.
(Shrestra 1998; Oleg and Stainton 2000; Manandhar 2002; Press et al. 2000), KATH 2013;
TUCH 2013; TPL 2013; eFloras 2013; Tropicos 2013).

Key: Distribution information is abbreviated by E (Eastern), C (Central), and W (West)
based on Nepal's three vegetative zones. N/A denotes where information is not available.

S.No.	Species	Family	Distribution	Altitude (m)	Habit	Host	Flowering Time	Fruiting Time
1	***Burmannia disticha*** L.	Burmanniaceae	C.E	1500 - 2600	Mycoheterotroph	N/A	April - June	June - September
2	***Burmannia nepalensis*** (Miers) Hook.f.	Burmanniaceae	E.	1600	Mycoheterotroph	N/A	July - December	July - December.
3	***Monotropa hypopithys*** L.	Ericaceae	W.C.	2400 - 3700	Mycoheterotroph	Fagaceae, Pinaceae	July - August	September - October
4	***Monotropa uniflora*** L.	Ericaceae	C.E.	900 - 3800	Mycoheterotroph	Fagaceae, Pinaceae	August - October	October - November
5	***Monotropastrum humile*** (D. Don) H. Hara	Ericaceae	C.E.	2200 - 3200	Mycoheterotroph	N/A	April - August	May - September
6	***Exacum nanum*** Klack.	Gentianaceae	E.	1700	Mycoheterotroph	N/A	N/A	N/A
7	***Eulophia bicallosa*** (D. Don) Hunt & Summerhayes	Orchidaceae	C.	2000 - 2500	Mycoheterotroph	N/A	June	June
8	***Eulophia dabia*** (D. Don) Hochreutiner	Orchidaceae	W.C.	2000	Mycoheterotroph	N/A	April - May	May - June
9	***Eulophia explanata*** Lindl.	Orchidaceae	C.	1200	Mycoheterotroph	N/A	N/A	N/A
10	***Eulophia flava*** (Lindl.) Hook.f.	Orchidaceae	C.	400	Mycoheterotroph	N/A	April - June	June
11	***Eulophia graminea*** Lindl.	Orchidaceae	C.	900 - 2100	Mycoheterotroph	N/A	April - May	May - June
12	***Eulophia spectabilis*** (Dennst.) Suresh	Orchidaceae	C.E.	450 - 1700.	Mycoheterotroph	N/A	April - June	April - June
13	***Galeola lindleyana*** (Hook.f & Thomson) Rchb.f.	Orchidaceae	E.	1800 - 2800	Mycoheterotroph	N/A	May - August	September - October
14	***Neottia acuminata*** Schltr.	Orchidaceae	W.C.	3200 - 3400	Mycoheterotroph	N/A	June - August	September - October
15	***Neottia listeroides*** Lindl.	Orchidaceae	W.C.	3000 - 3200	Mycoheterotroph	N/A	July - September	September - October
16	***Cephalanthera longifolia*** (L.) Fritsch	Orchidaceae	W.C	1200 - 3200	Mycoheterotroph	N/A	May - June	September - Octoer
17	***Epipogium aphyllum*** Sw.	Orchidaceae	W.C	3000 - 3400	Mycoheterotroph	N/A	August - September	October
18	***Epipogium roseum*** (D.Don) Lindl.	Orchidaceae	C.	1200 - 3600	Mycoheterotroph	N/A	August - September	October
