# Supplementary material for: An ethnobotanical analysis of parasitic plants (Parijibi) in the Nepal Himalaya
Source: J Ethnobiol Ethnomed. 2016 Feb 24;12:14. doi: 10.1186/s13002-016-0086-y (PMC4765049; doi:10.1186/s13002-016-0086-y)
Supplement: Additional file 3: — Plant distribution maps of parasitic and mycoheterotropic plant species found in Nepal. (PDF 106616 kb) [file 13002_2016_86_MOESM3_ESM.pdf]

Appendix III. Distribution of parasitic and mycoheterotrophic plant species in Nepal, based on our study, literature review and specimen available from TUCH (Kirtipur, Kathmandu) and KATH (Godawari, Lalitpur).

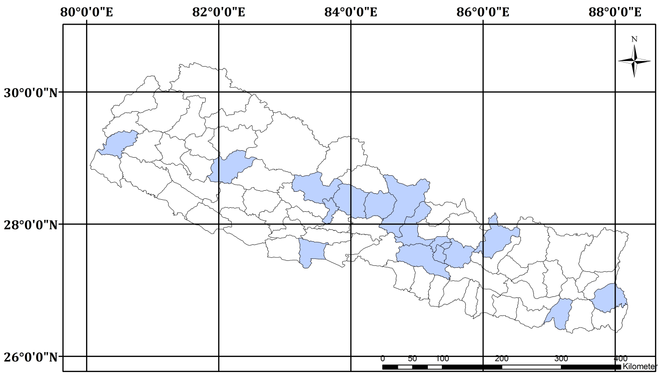

Map 1. *Aeginetia indica* L.
(Orobanchaceae)

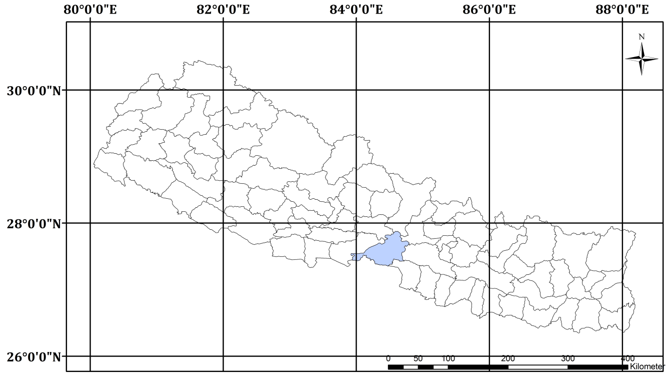

Map 2. *Aeginetia pedunculata* (Roxb.) Wall
(Orobanchaceae)

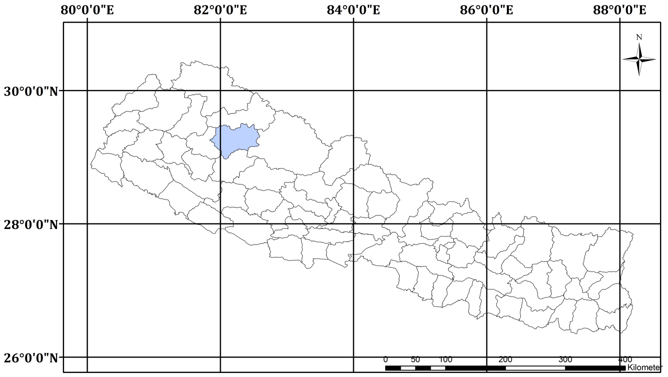

Map 3. *Balanophora involucrata* Hook.
(Balanophoraceae)

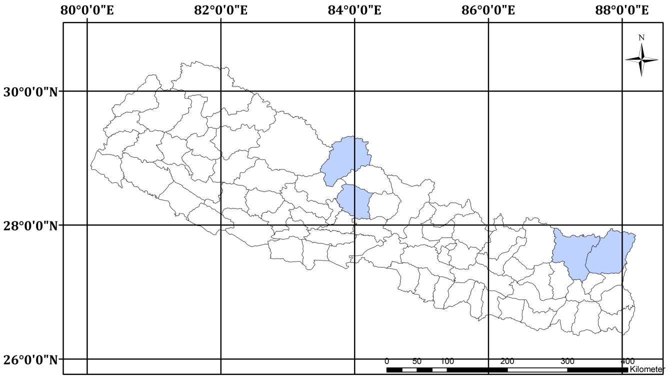

Map 4. *Balanophora polyandra* Griffith.
(Balanophoraceae)

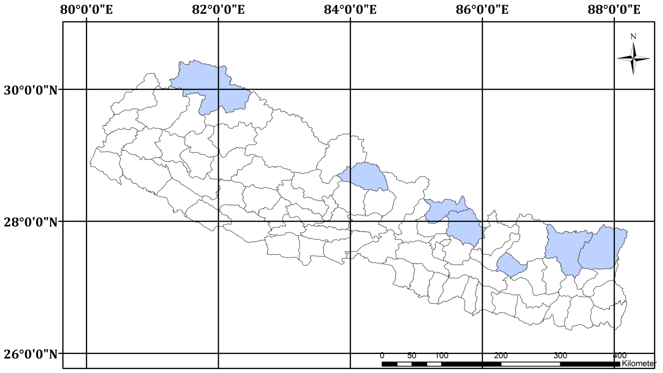

Map 5. *Boschniakia himalaica* Hook & Thomson ex Hook.
(Orobanchaceae)

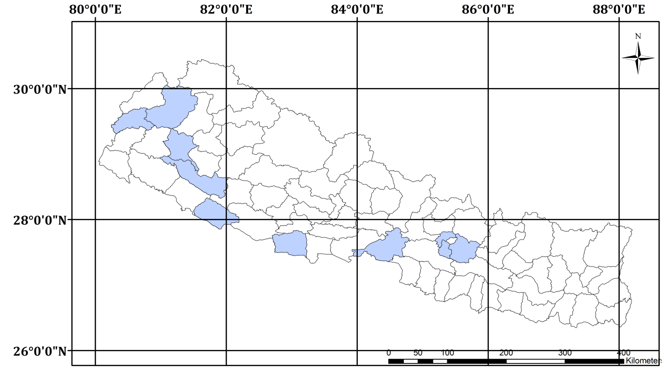

Map 6. *Centranthera cochinchinensis* var. *nepalensis* (D.Don) Merr.
(Orobanchaceae)

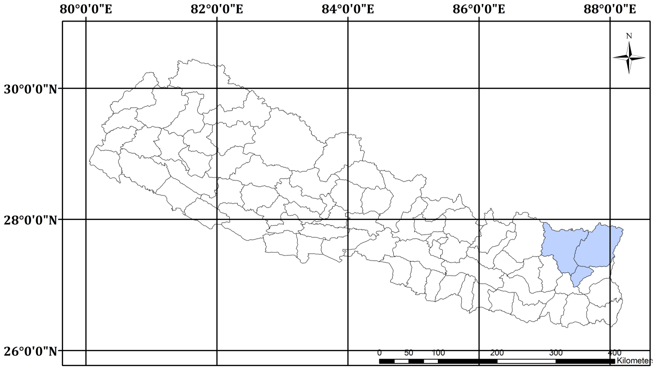

Map 7. *Centranthera grandiflora* Benth. (Orobanchaceae)

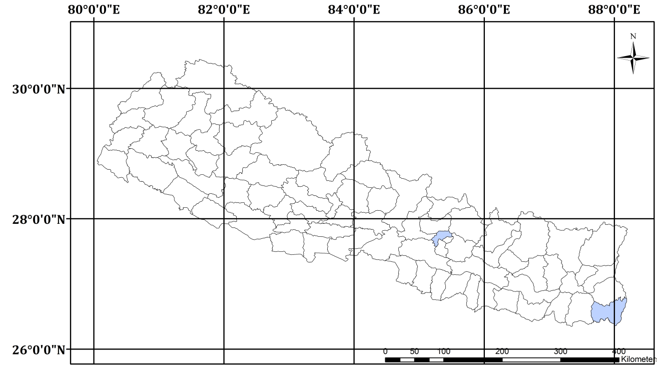

Map 8. *Cuscuta chinensis* Lam. (Convolvulaceae)

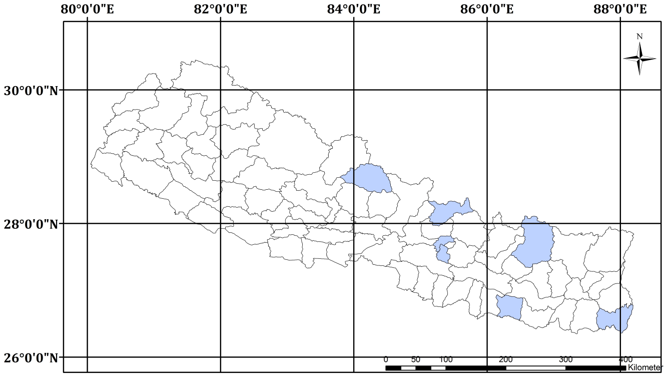

Map 9. *Cuscuta europaea* Engelm. (Convolvulaceae)

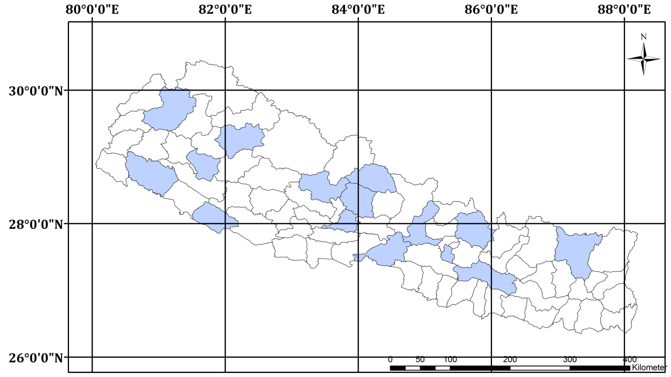

Map 10. *Cuscuta reflexa* Roxb. (Convolvulaceae)

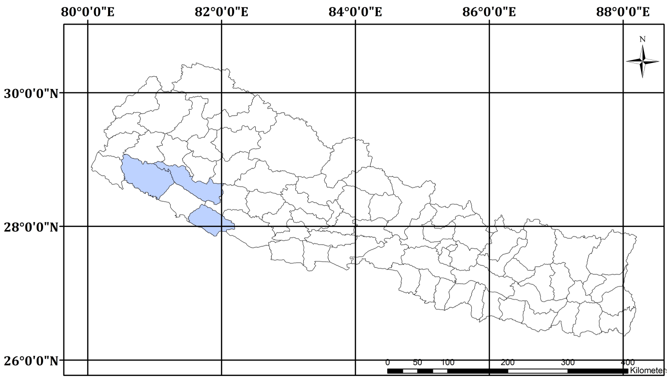

Map 11. *Dendrophthoe falcata* (L.F.) Etting. (Loranthaceae)

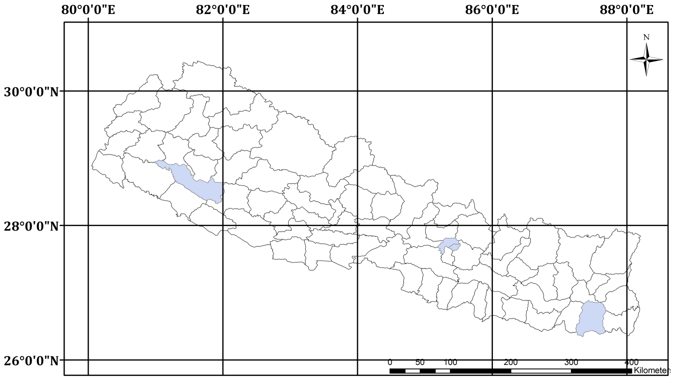

Map 12. *Dendrophthoe petandra* (L.) Miq. (Loranthaceae)

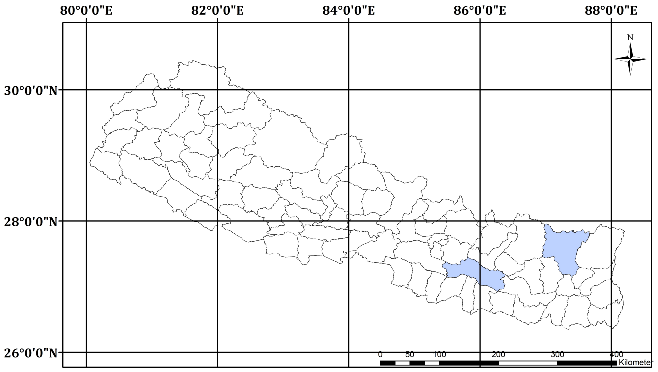

Map 13. *Drufrenoya platyphylla* (Spreng.) Stauf.
(Amphorogynaceae)

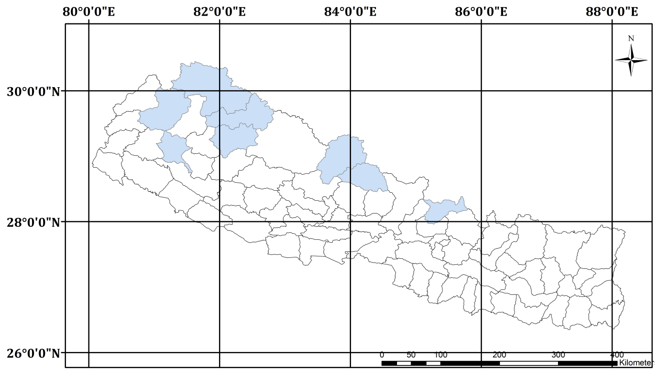

Map 14. *Euphrasia himalayica* Wettst.
(Orobanchaceae)

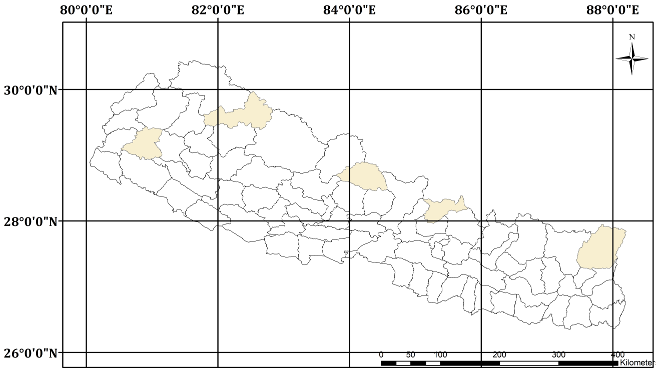

May 15. *Euphrasia platyphylla* Penn.
(Orobanchaceae)

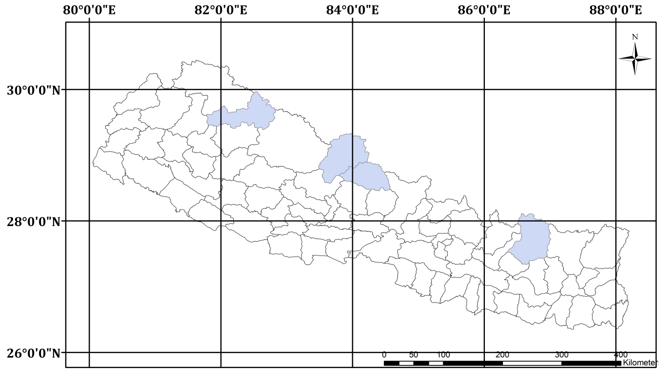

Map 16. *Euphrasia schlagintweitii* Wettst.
(Orobanchaceae)

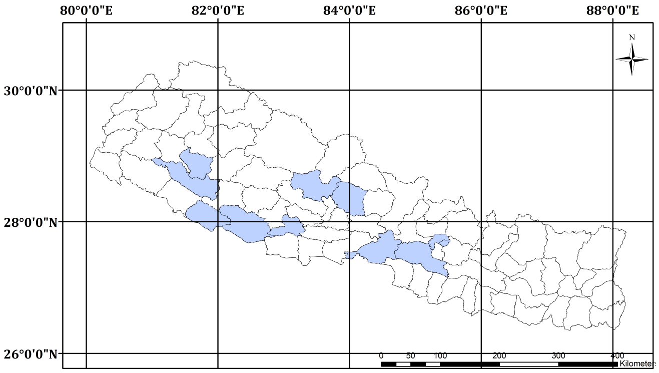

Map 17. *Helixanthera ligustrina* (Wall. Danser)
(Loranthaceae)

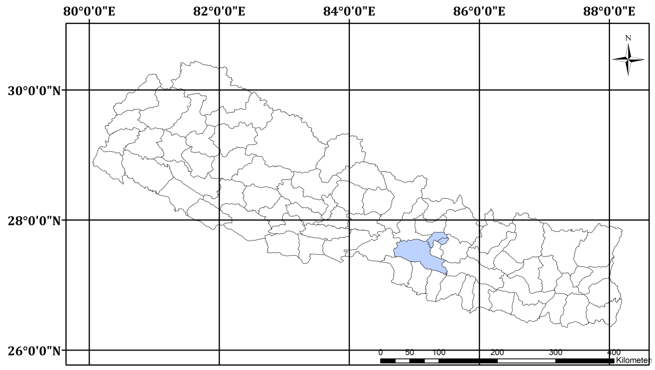

Map 18. Helixanthera parasitica Lour.
(Loranthaceae)

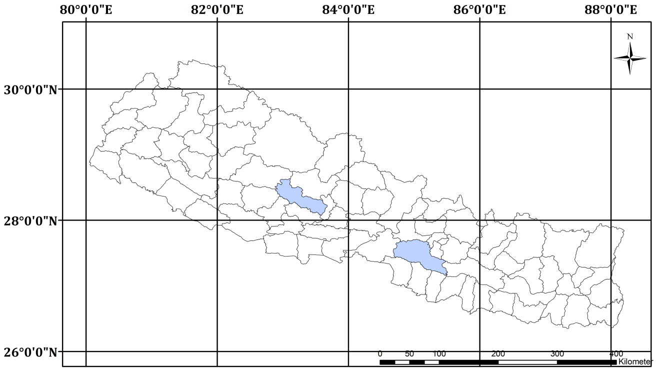

Map 19. *Korthalsella japonica* (Thunb.) Engl.
(Loranthaceae)

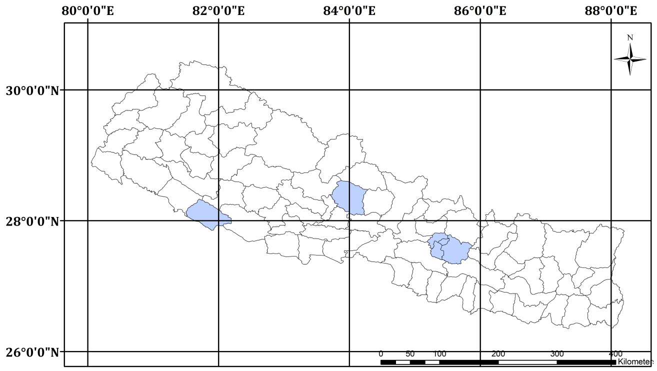

Map 20. *Loranthus odoratus* Wall.
(Loranthaceae)

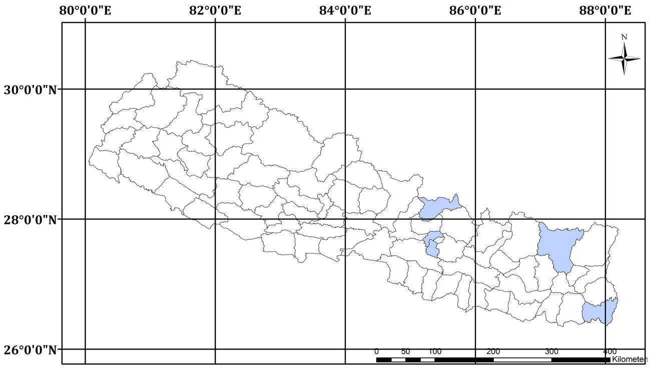

Map 21. *Macrosolen cochinchinensis* (Lour.) Tiegh
(Loranthaceae)

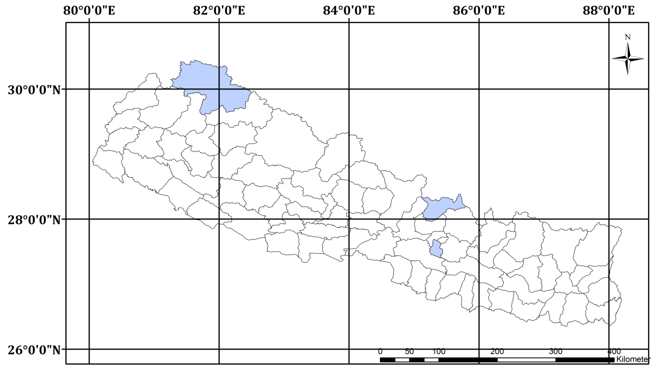

Map 22. *Monotropa uniflora* L.
(Ericaceae)

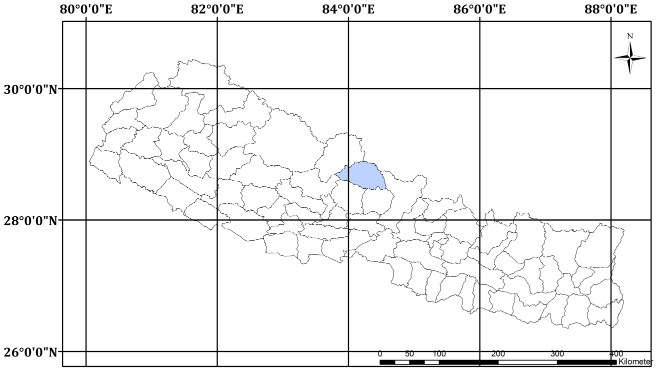

Map 23. *Monotropa hypopithys* L.
(Ericaceae)

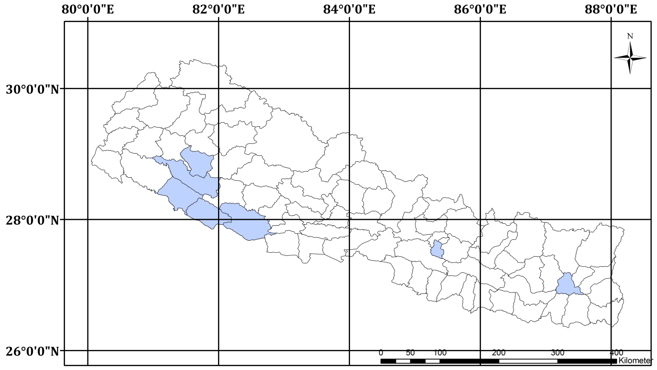

Map 24. *Monotropastrum humile* (D.Don) Hunt & Summerhayes
(Ericaceae)

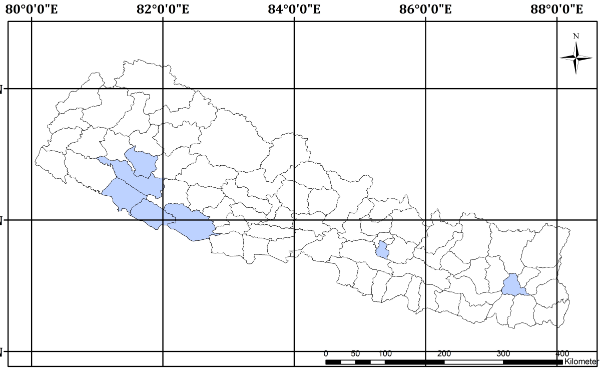

Map 25. *Orobanche aegyptiaca* Pers.
(Orobanchaceae)

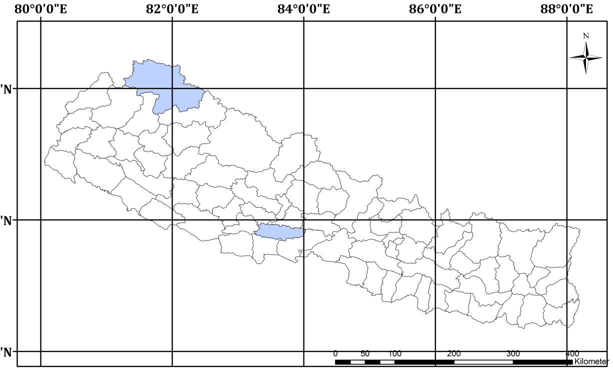

Map 26. *Orobanche alba* Steph. Ex Willd.
(Orobanchaceae)

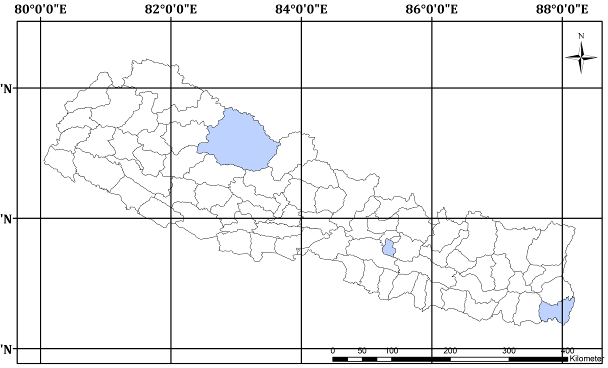

Map 27. *Orobanche coeruescens* Steph.
(Orobanchaceae)

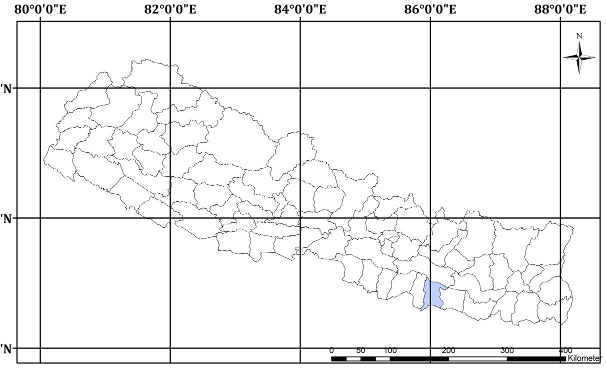

Map 28 *Orobanche solmsii* C.C. Clarke ex Hook. f.
(Orobanchaceae)

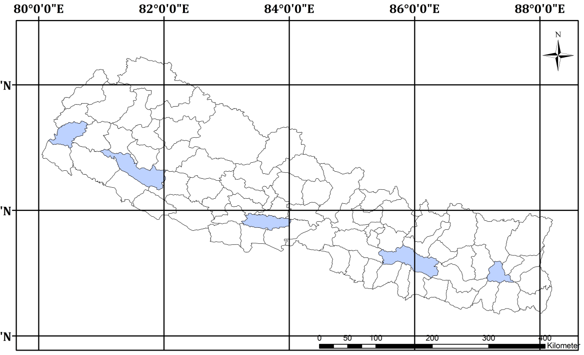

Map 29. *Osyris arborea* Salzm. ex Decene.
(Santalaceae)

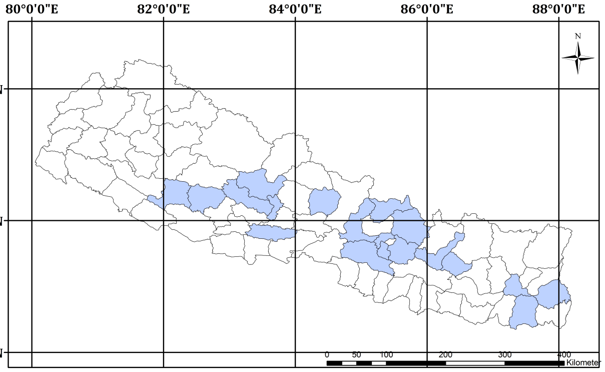

Map 30. *Osyris wightiana* Salzm. ex Decene.
(Santalaceae)

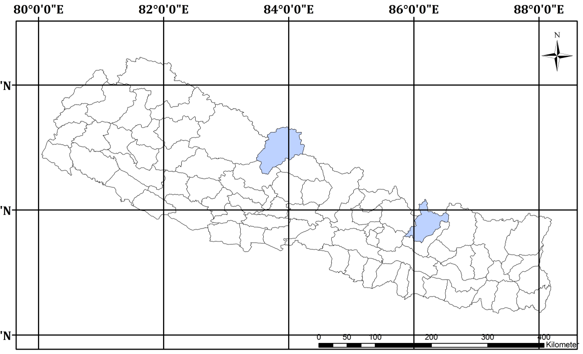

Map 31. *Pedicularis anserantha* T. Yamaz var. *elevantogaleta* (Orobanchaceae)

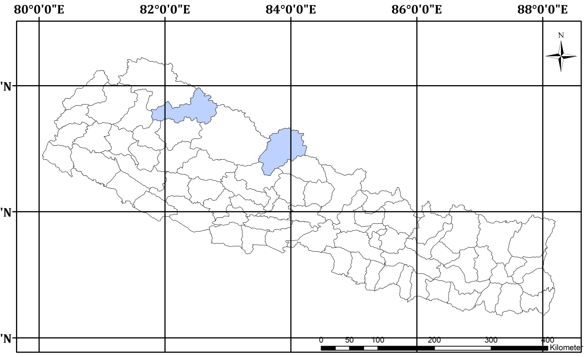

Map 32. *Pedicularis anserantha* T. Yamaz var. *anserantha* (Orobanchaceae)

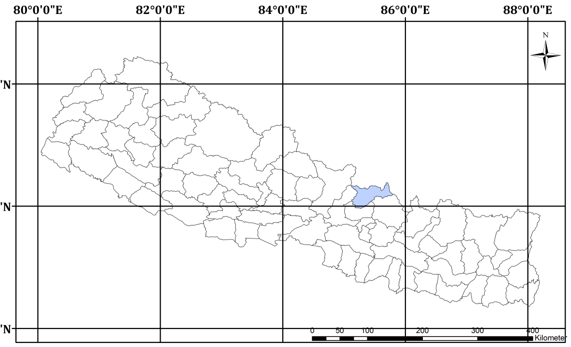

Map 33. *Pedicularis albiflora* (Hook f.) Prain (Orobanchaceae)

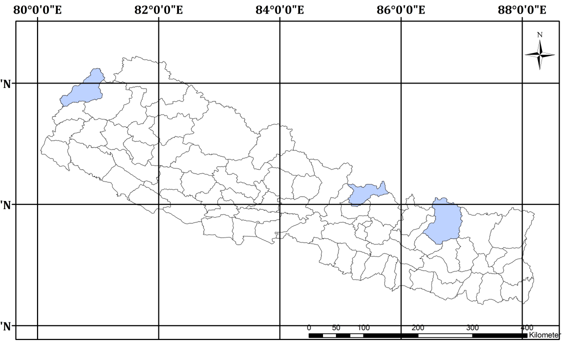

Map 34. *Pedicularis bifida* (Buch.-Ham. Ex D.Don) Penn. (Orobanchaceae)

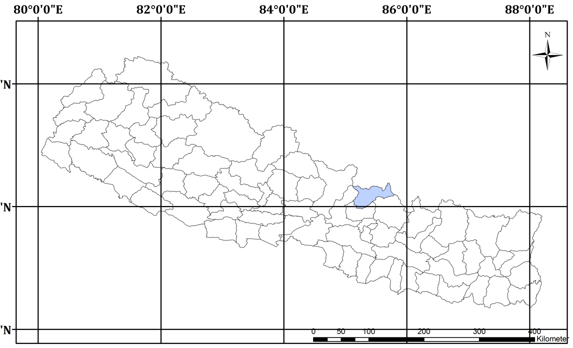

Map 35. *Pedicularis brevifolia* D. Don. (Orobanchaceae)

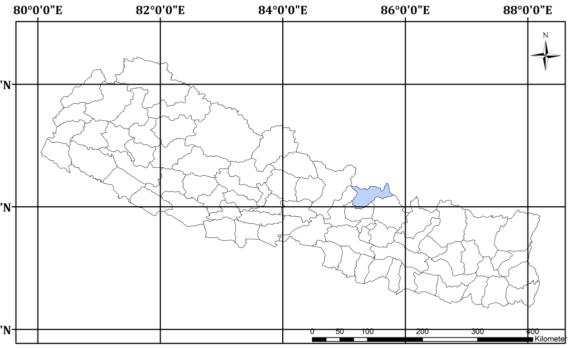

Map 36. *Pedicularis chamissonoides* T. Yamaz (Orobanchaceae)

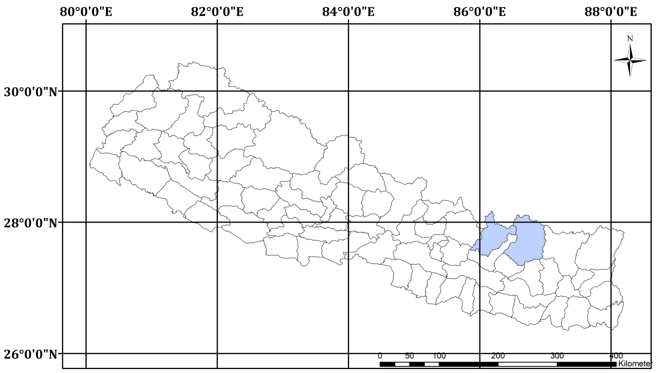

Map 37. *Pedicularis clarkei* Hook.
(Orobanchaceae)

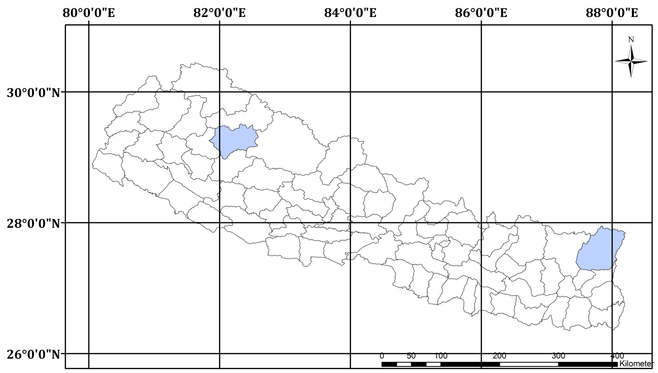

Map 38. *Pedicularis collata* Prain.
(Orobanchaceae)

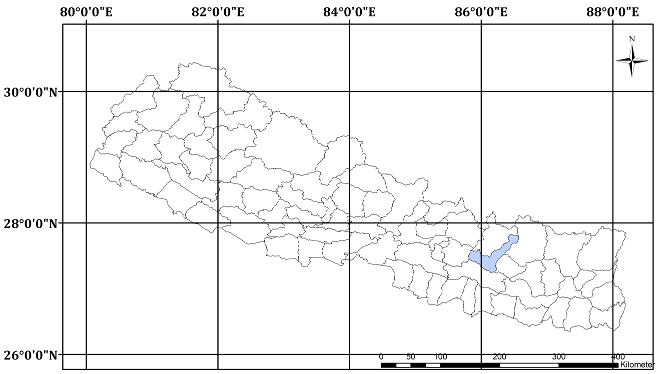

Map 39. *Pedicularis confertiflora* Prain.
(Orobanchaceae)

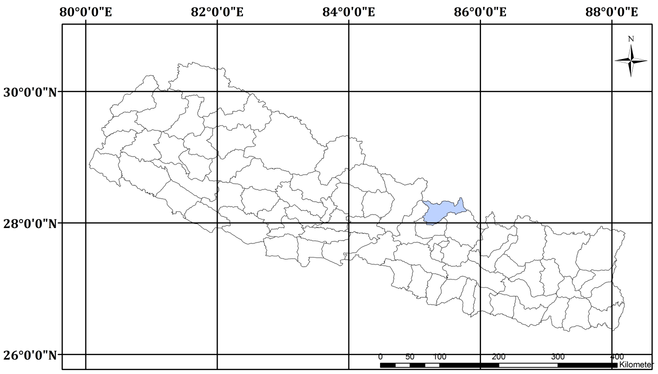

Map 40. *Pedicularis denudata* Hook. f.
(Orobanchaceae)

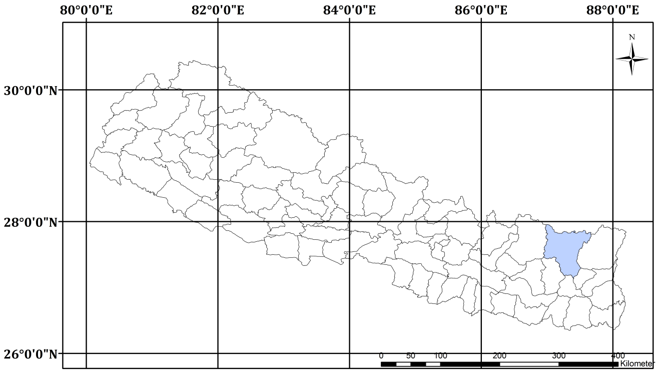

Map 41. *Pedicularis diffusa* Prian.
(Orobanchaceae)

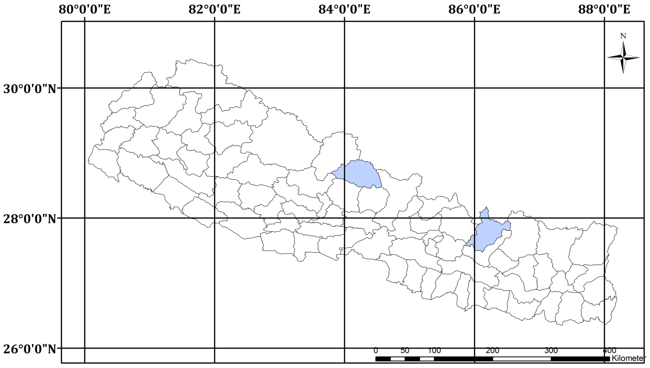

Map 42. *Pedicularis elwesii* Hook. f.
(Orobanchaceae)

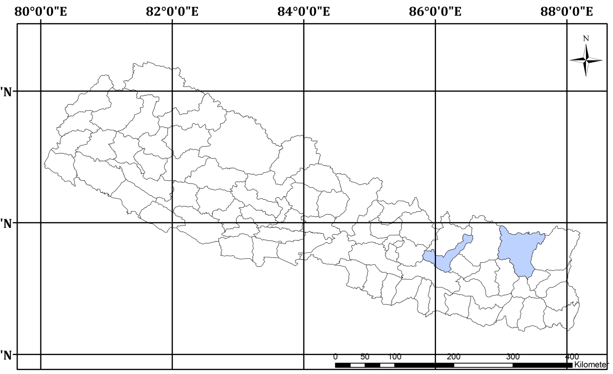

Map 43. *Pedicularis flexuosa* Hook. f.
(Orobanchaceae)

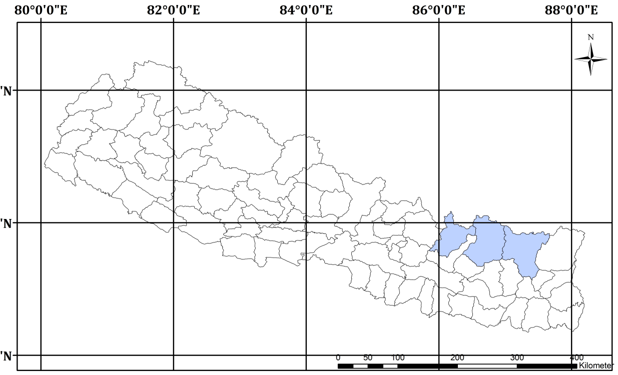

Map 44. *Pedicularis furfuracea* Wall. Ex. Benth.
(Orobanchaceae)

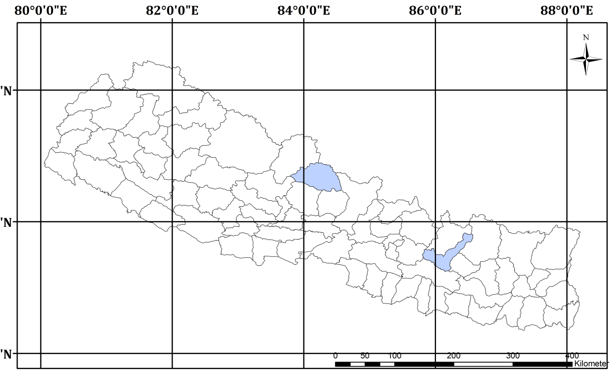

Map 45. *Pedicularis globifera* Hook f.
(Orobanchaceae)

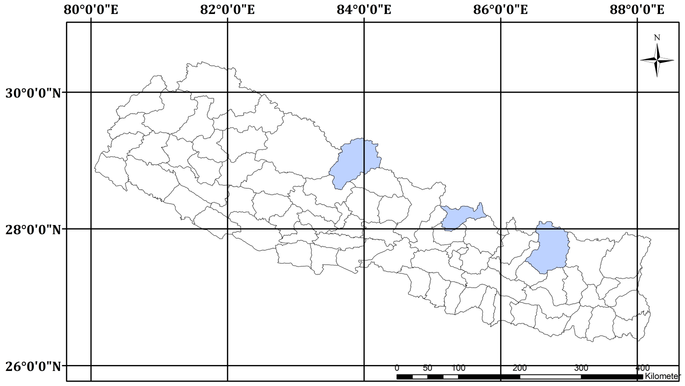

Map 46. *Pedicularis gracilis* Wall. ex Benth. Subsp. *gracilis*
(Orobanchaceae)

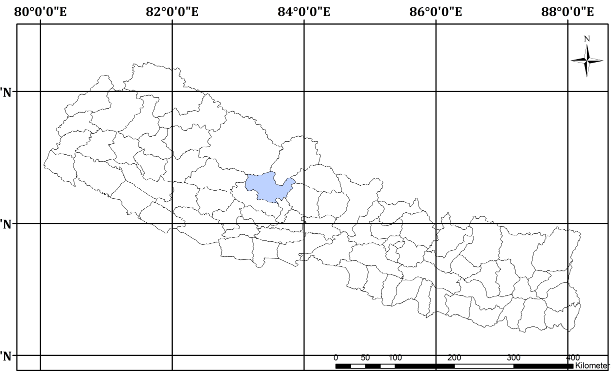

Map 47. *Pedicularis gracilis* Wall. ex Benth. Subsp. *macrocarpa*
(Orobanchaceae)

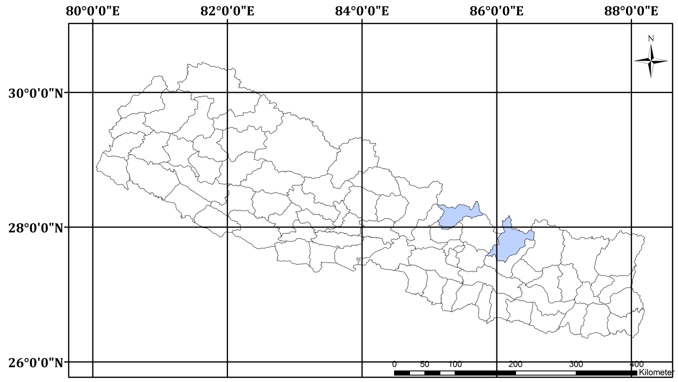

Map 48. *Pedicularis heydeii* Prain.
(Orobanchaceae)

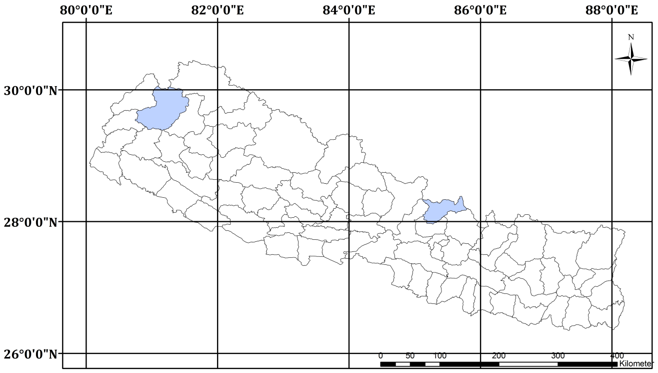

Map 49. *Pedicularis hoffmeisteri* Klotzsch.
(Orobanchaceae)

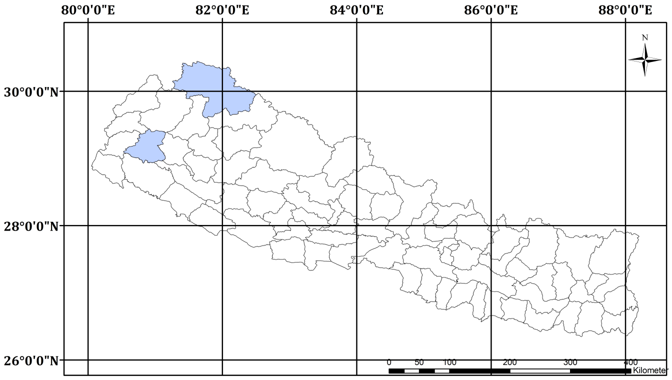

Map 50. *Pedicularis hookeriana* Wall.
(Orobanchaceae)

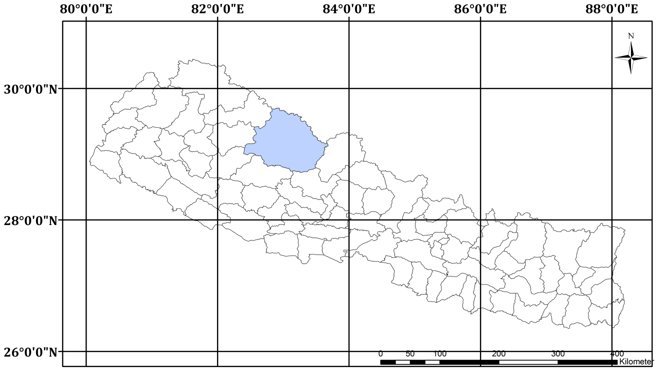

Map 51. *Pedicularis instar* Prain ex Maxim.
(Orobanchaceae)

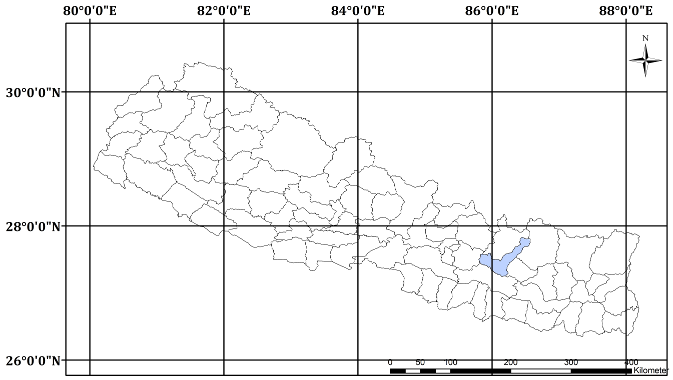

Map 52. *Pedicularis intergrifolia* Hook. f.
(Orobanchaceae)

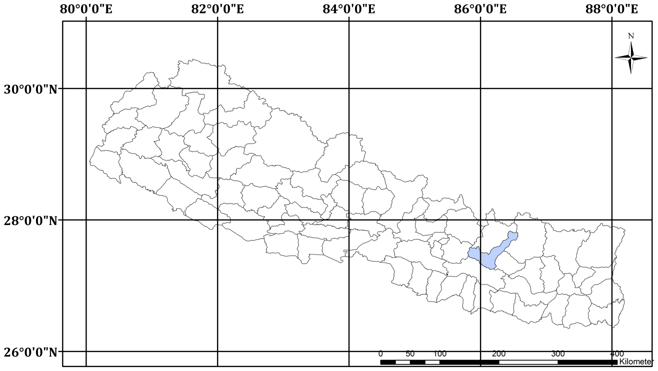

Map 53. *Pedicularis kansuensis* Maxim.
(Orobanchaceae)

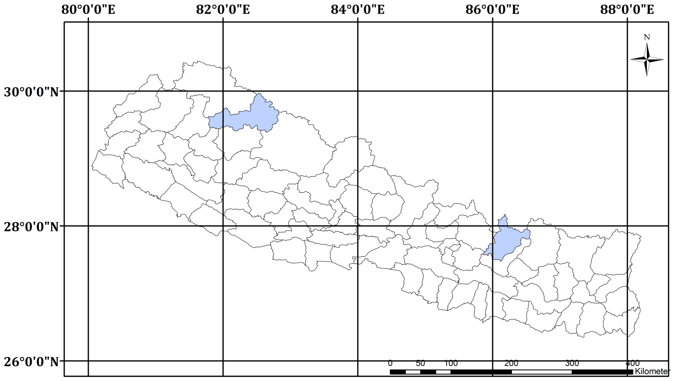

Map 54. *Pedicularis klotzschii* Hurus.
(Orobanchaceae)

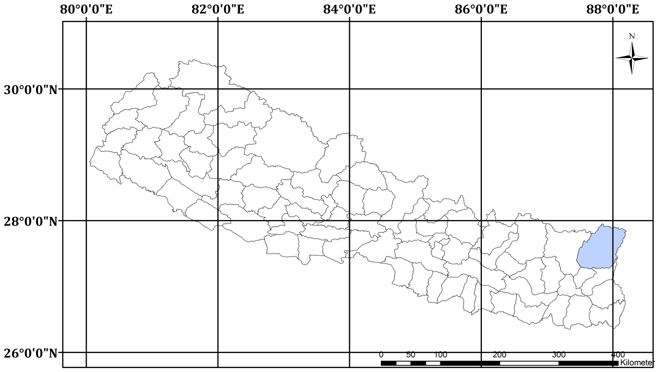

Map 55. *Pedicularis lachnoglossa* Hook. f
(Orobanchaceae)

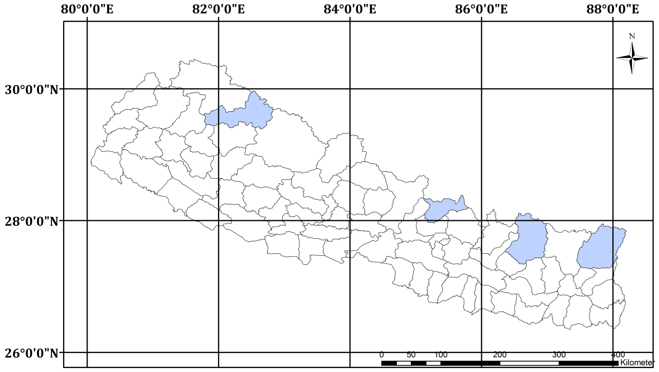

Map 56. *Pedicularis longiflora* Rudolph subsp. *tubiformis* (Klotzsch.) Tsoong.
(Orobanchaceae)

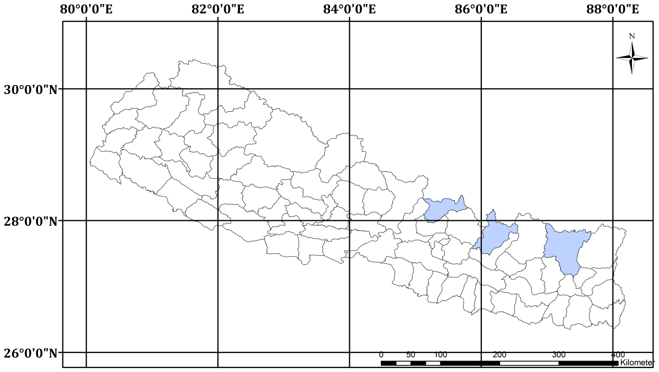

Map 57. *Pedicularis megalantha* D. Don.
(Orobanchaceae)

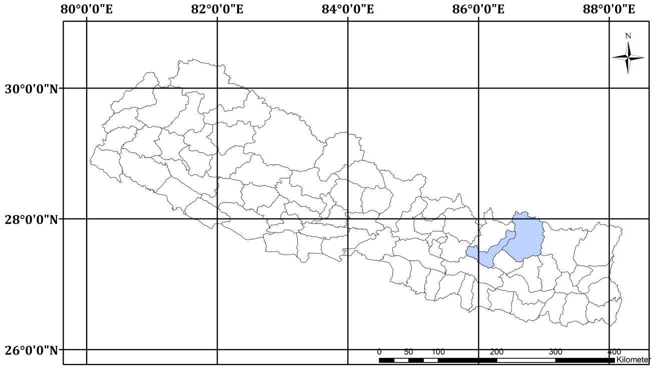

Map 58. *Pedicularis megalochila* Li. subsp. *longituba*
(Orobanchaceae)

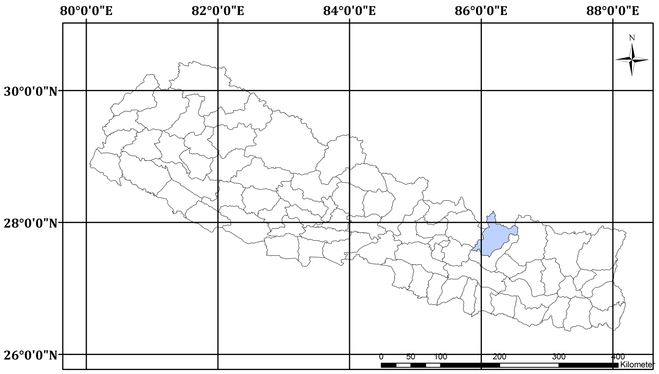

Map 59. *Pedicularis microcalyx* Hook. f.
(Orobanchaceae)

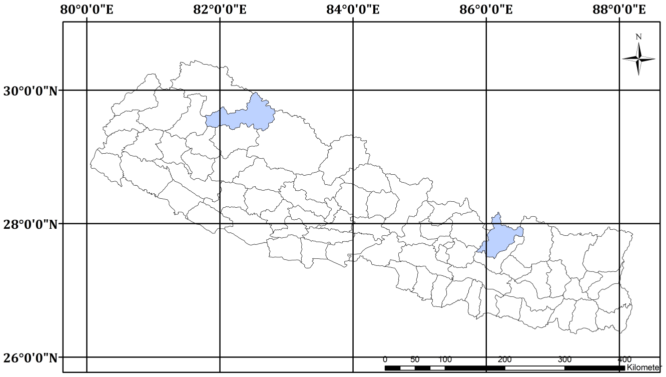

Map 60. *Pedicularis mollis* Wall. ex Benth.
(Orobanchaceae)

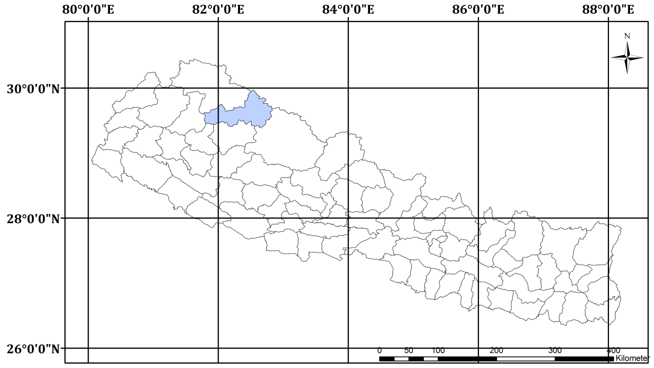

Map 61. *Pedicularis muguensis* T. Yamaz
(Orobanchaceae)

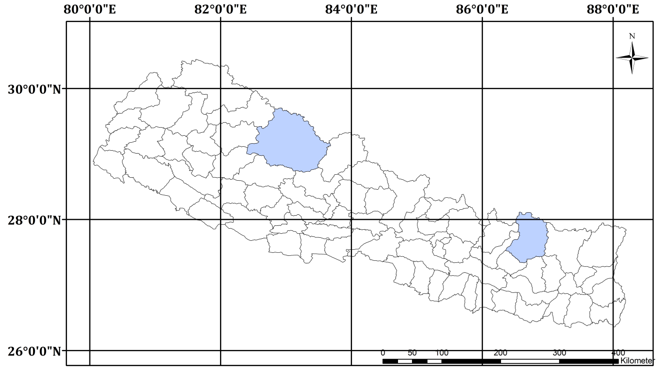

Map 62. *Pedicularis muscoides* H.L. Li
(Orobanchaceae)

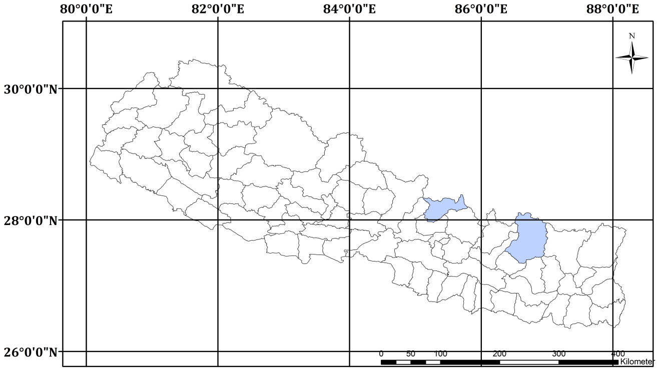

Map 63. *Pedicularis nepalensis* Prain.
(Orobanchaceae)

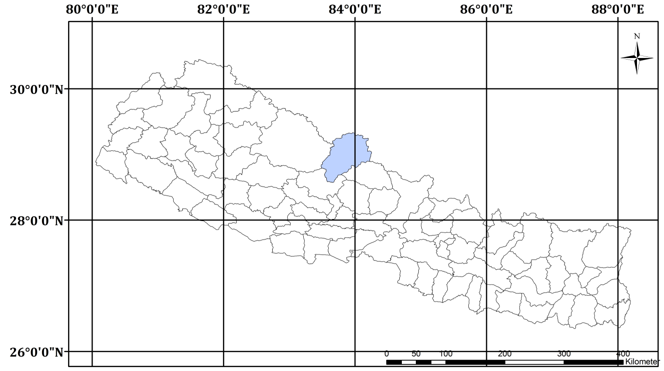

Map 64. *Pedicularis nodosa* Penn.
(Orobanchaceae)

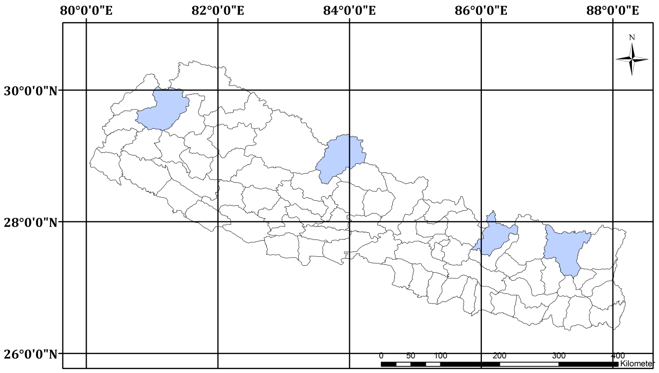

Map 65. *Pedicularis oederi* subsp. *oederi* var. *heteroglossa* Vahl.
(Orobanchaceae)

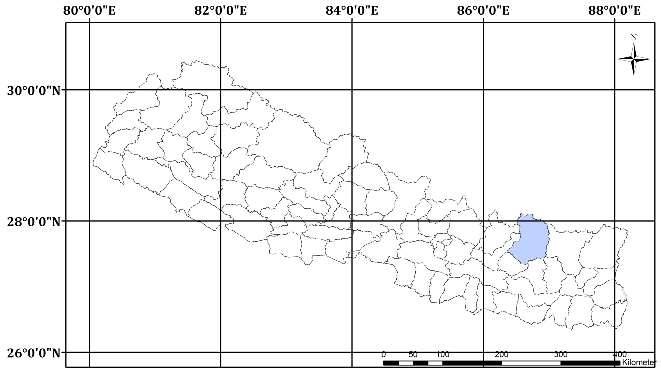

Map 66. *Pedicularis pauciflora* (Prain.) Penn.
(Orobanchaceae)

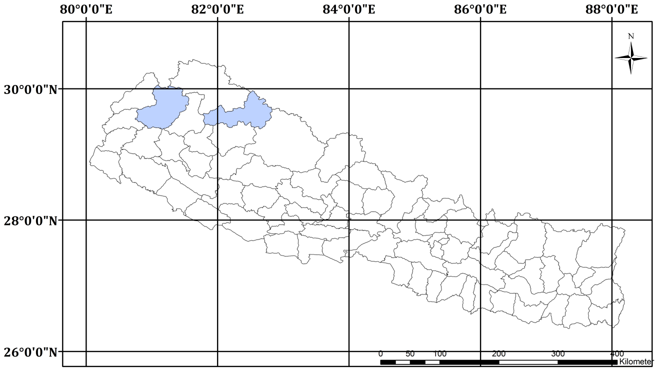

Map 67. *Pedicularis pectinata* Wall. ex. Beth.
(Orobanchaceae)

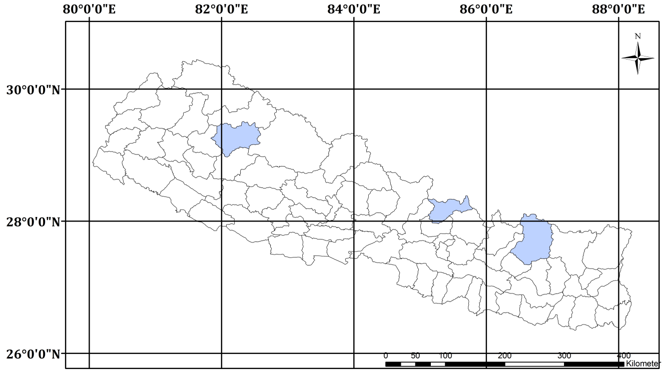

Map 68. *Pedicularis porrecta* Wall.
(Orobanchaceae)

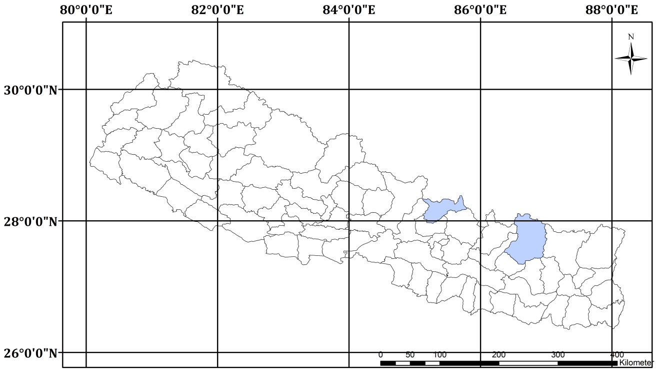

Map 69. *Pedicularis pseudoregeliana* Tsoong.
(Orobanchaceae)

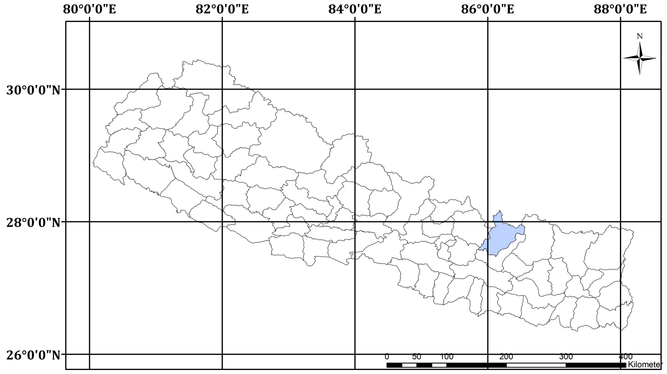

Map 70. *Pedicularis regeliana* Prain.
(Orobanchaceae)

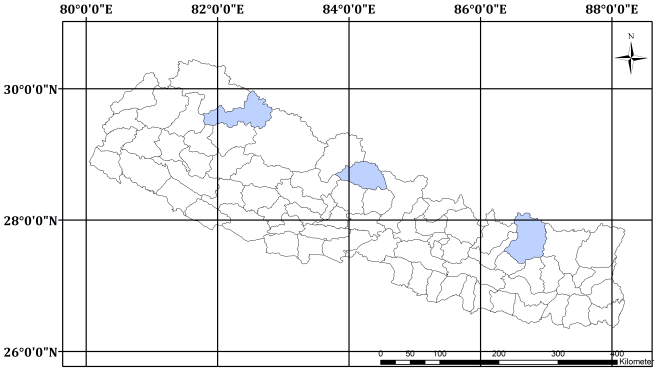

Map 71. *Pedicularis rhianthoides* Schrenk. subsp. *labellata* (Jacquem.) Prain. ex Penn.
(Orobanchaceae)

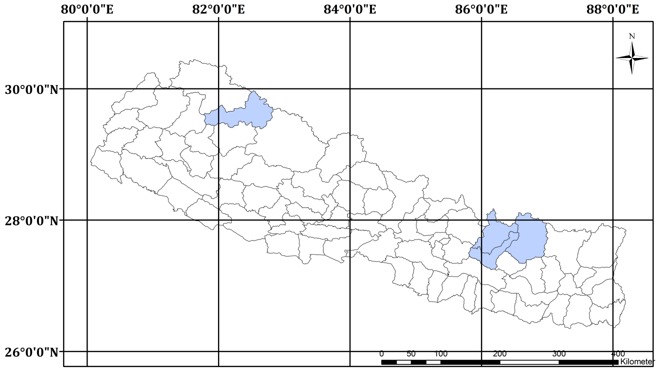

Map 72. *Pedicularis roylei* Tsoong.
(Orobanchaceae)

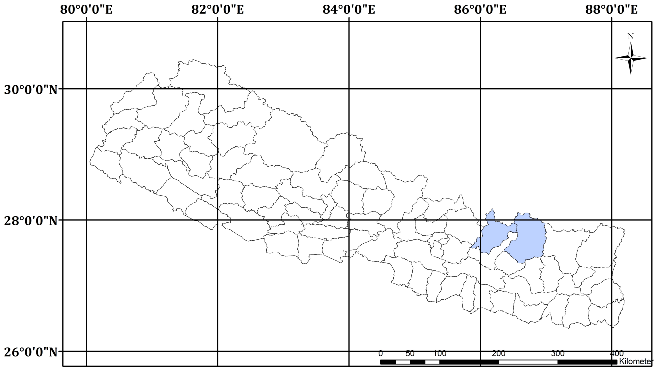

Map 73. *Pedicularis schizorrhyncha* Prain.
(Orobanchaceae)

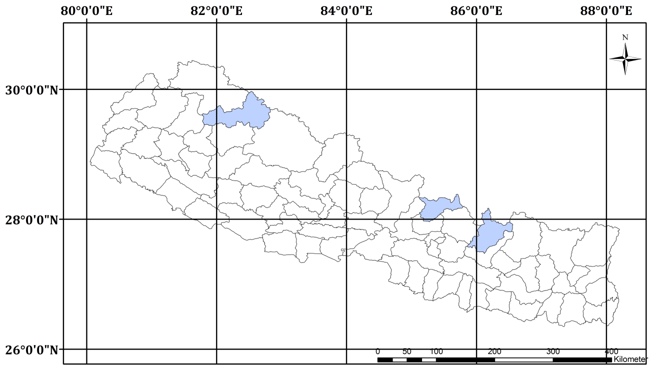

Map 74. *Pedicularis scullyana* Prain ex. Maxim.
(Orobanchaceae)

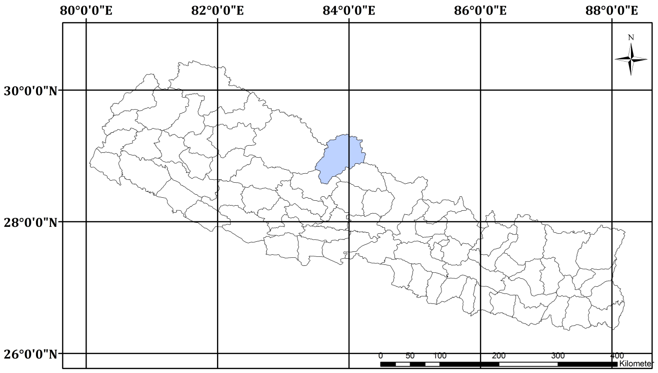

Map 75. *Pedicularis sectifolia* T. Yamaz.
(Orobanchaceae)

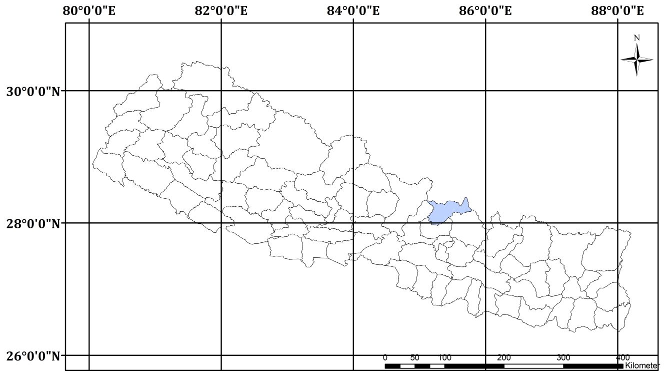

Map 76. Pedicularis sikkimensis Bonati
(Orobanchaceae)

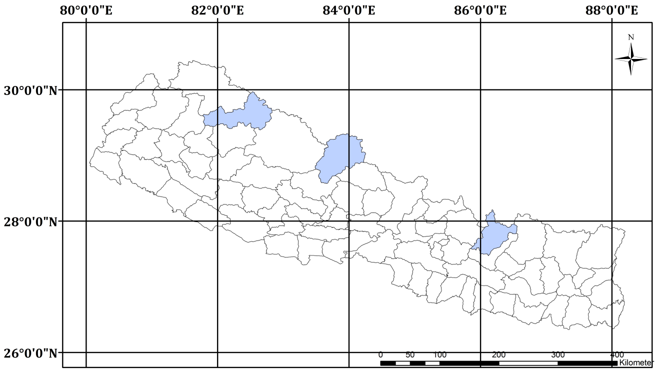

Map 77. *Pedicularis siphonantha* D. Don.
(Orobanchaceae)

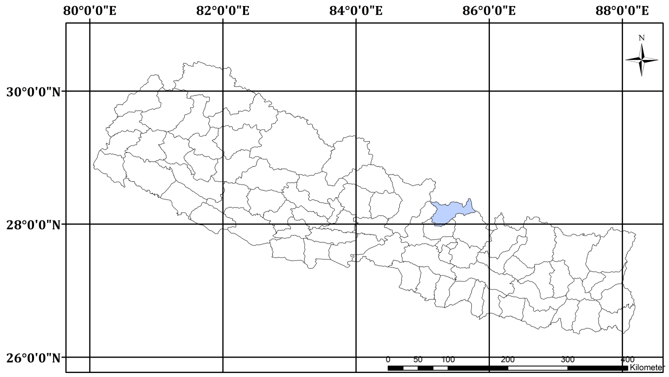

Map 78. *Pedicularis trichodonta* T. Yamaz.
(Orobanchaceae)

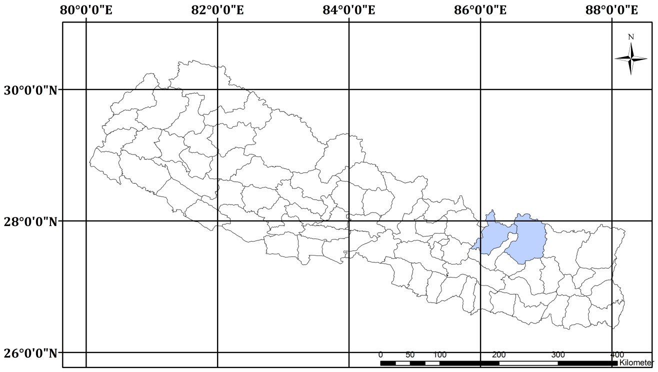

Map 79. *Pedicularis trichoglossa* Hook f.
(Orobanchaceae)

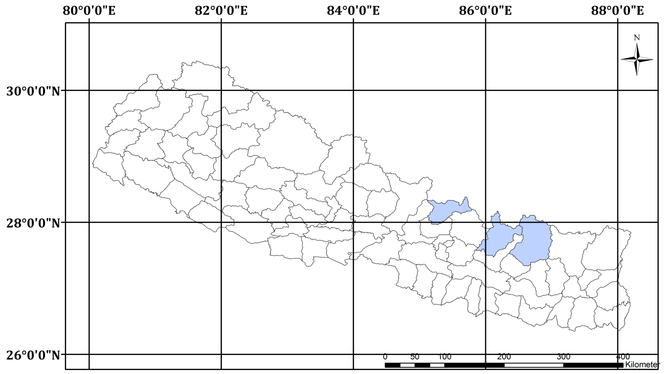

Map 80. *Pedicularis wallichii* Bunge
(Orobanchaceae)

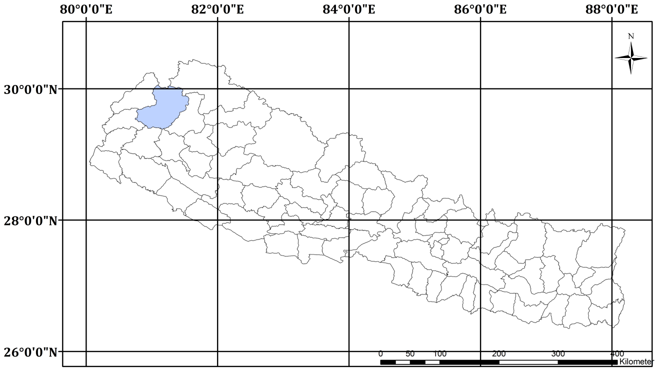

Map 81. *Pedicularis yamazakiana* R.R. Mill
(Orobanchaceae)

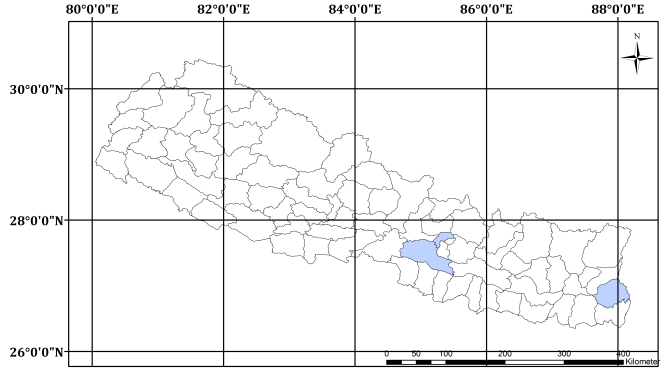

Map 82. *Pyrularia edulis* (Wallich.) A. Candolle.
(Cervantesiaceae)

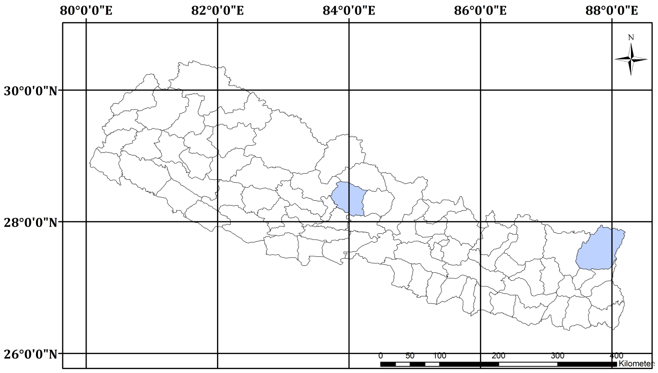

Map 83. *Rhopalocenmis phalloides* Jungh.
(Balanophoraceae)

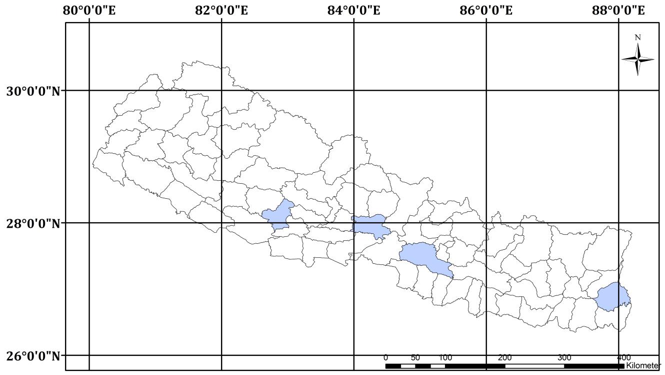

Map 84. *Santalum album* L.
(Santalaceae)

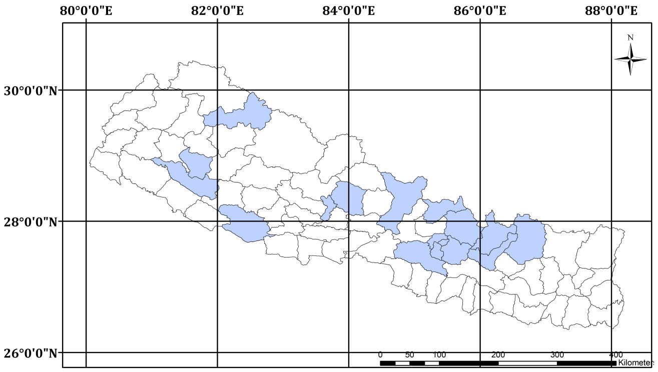

Map 85. *Scurrula elata* (Edgeworth.) Danser
(Loranthaceae)

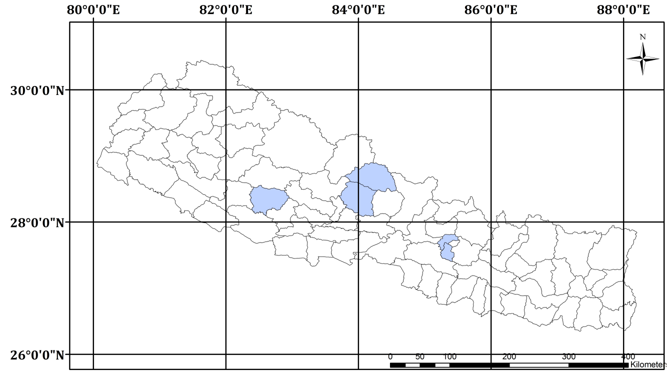

Map 86. *Scurulla parasitica* var. *graciliflora* (Roxb ex Schult) H.S. Kiu
(Loranthaceae)

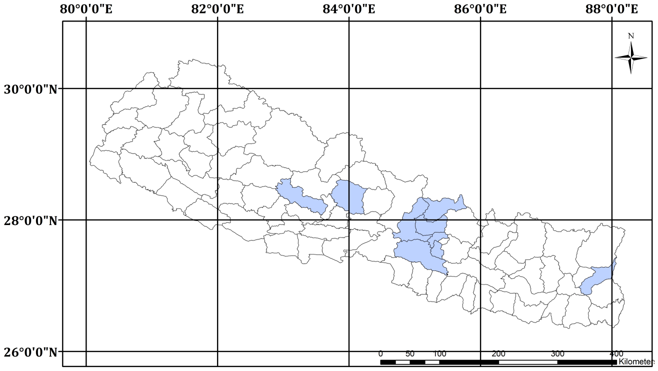

Map 87. *Scurulla parasitica* L.
(Loranthaceae)

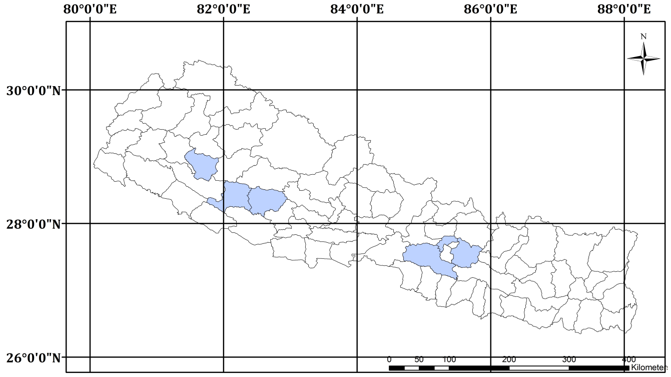

Map 88. *Scurulla pulverulenta* (Wall.) G. Don
(Loranthaceae)

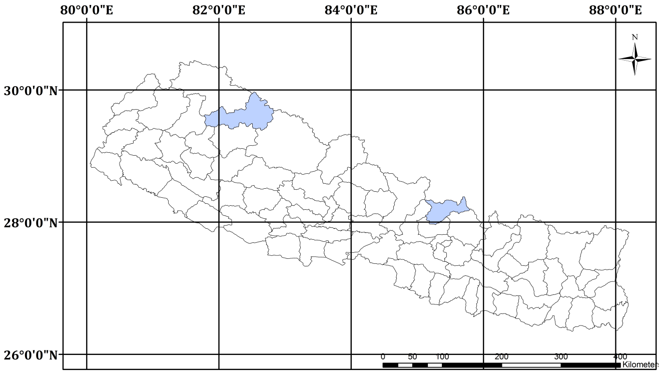

Map 89. *Striga asiatica* (L.) Kuntze.
(Orobanchaceae)

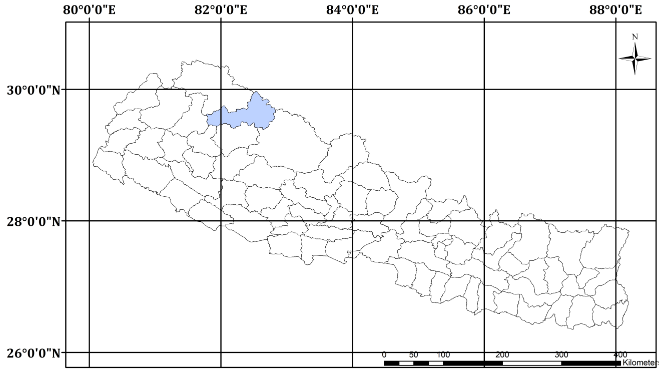

Map 90. *Striga gesneroides* (Willd.) Vatke
(Orobanchaceae)

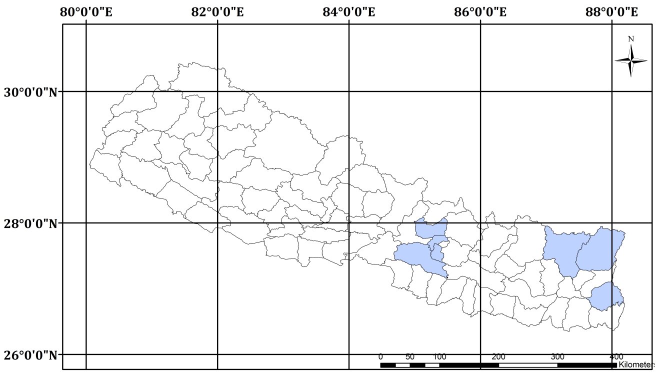

Map 91. *Taxillus umbelifera* (Schult.) G. Don.
(Loranthaceae)

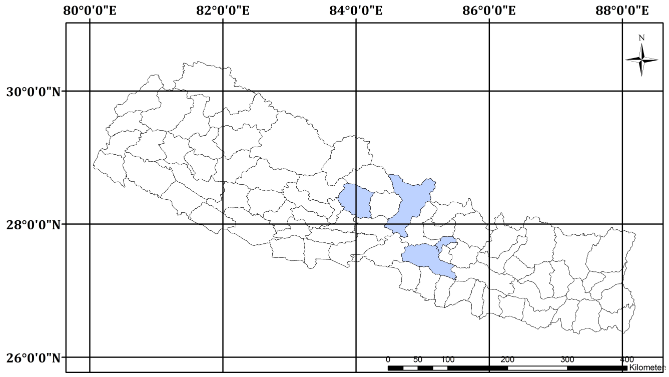

Map 92. *Taxillus vestitus* (Wall.) Danser
(Loranthaceae)

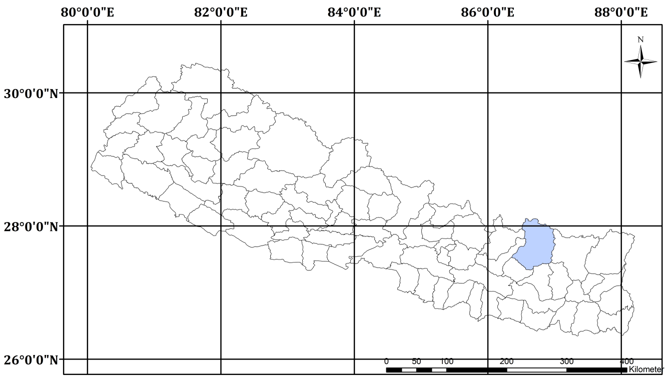

Map 93. *Thesium emodi* Hendrych
(Santalaceae)

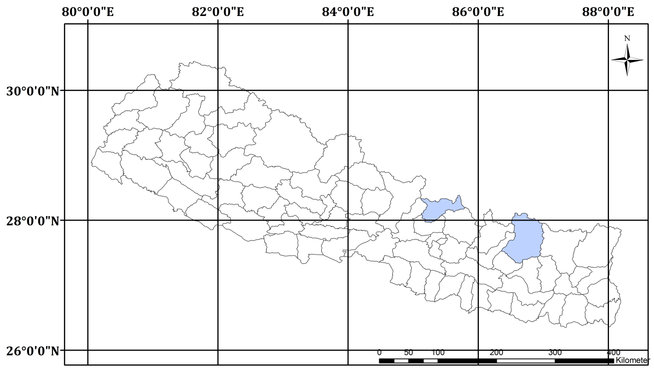

Map 94. *Thesium himalense* Royle
(Santalaceae)

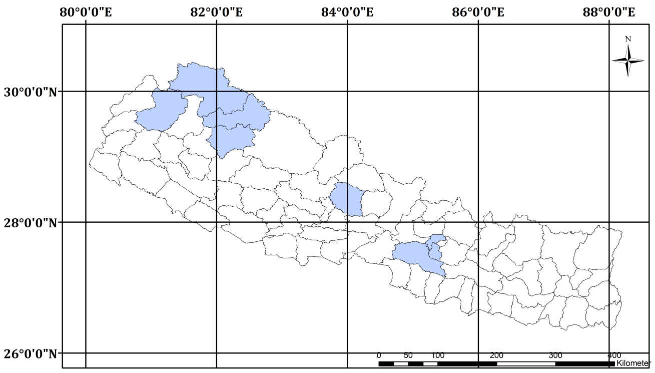

Map 95. *Viscum album* L.
(Viscaceae)

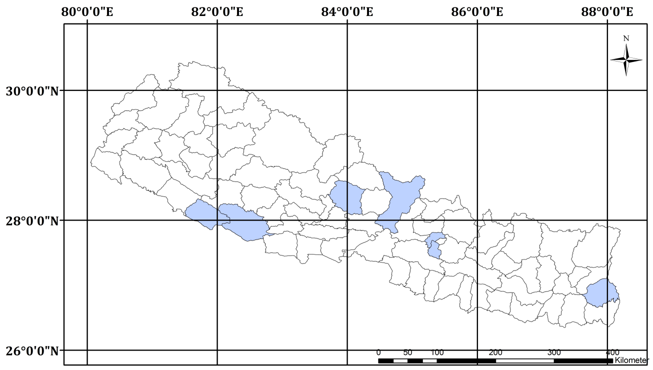

Map 96. *Viscum articulatum* var. *articulatum* Burm.
(Viscaceae)

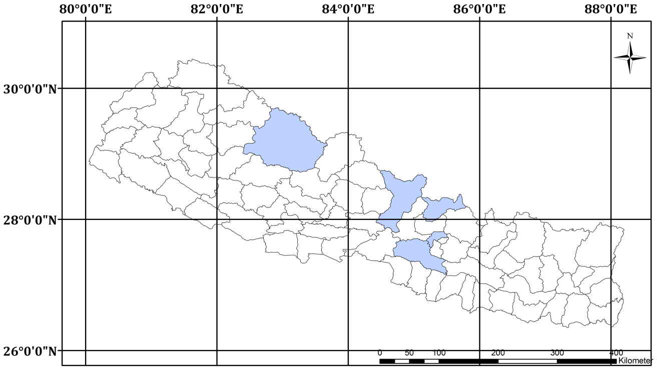

Map 97. *Viscum articulatum* var. *liquidambarcolum* Burm. f. (Viscaceae)

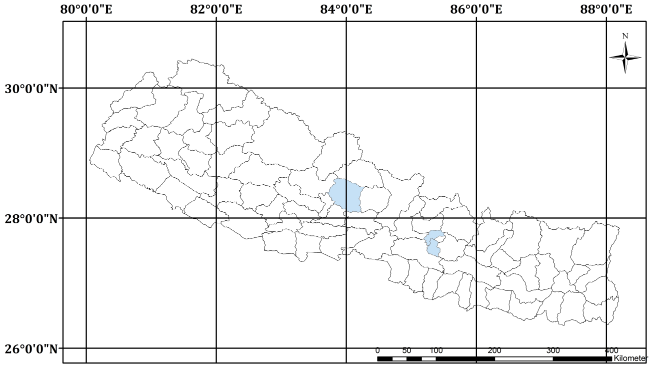

Map 98. *Viscum loranthii* Elmer. (Viscaceae)

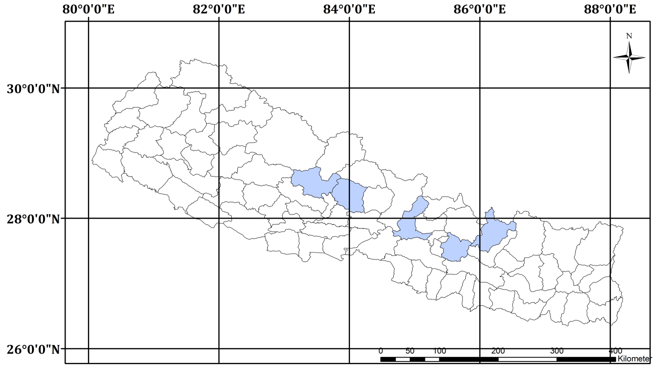

Map 99. *Viscum monoicum* Roxb. ex DC (Viscaceae)

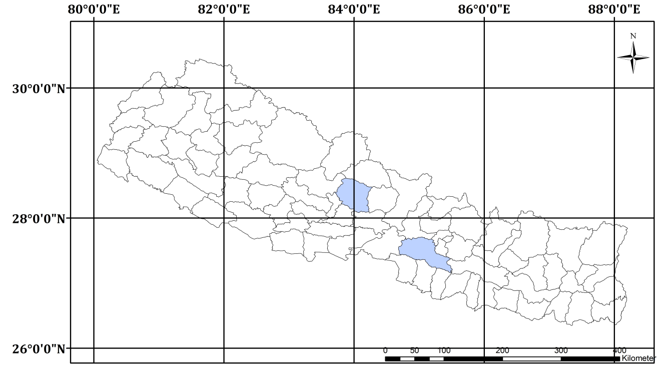

Map 100. Schopfia multiflora L. (Viscaceae)

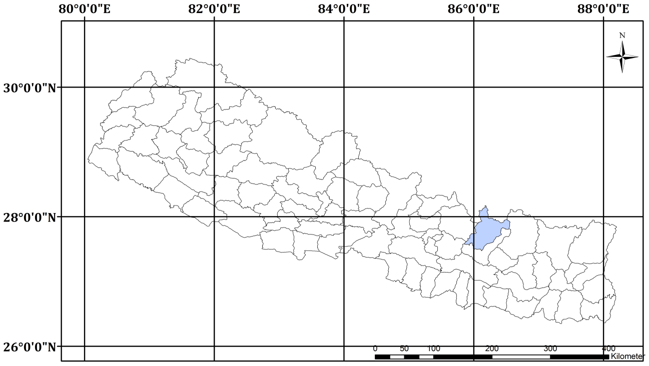

Map 101. *Viscum orientale* Willd. (Viscaceae)
