# Supplementary material for: An ethnobotanical analysis of parasitic plants (Parijibi) in the Nepal Himalaya
Source: J Ethnobiol Ethnomed. 2016 Feb 24;12:14. doi: 10.1186/s13002-016-0086-y (PMC4765049; doi:10.1186/s13002-016-0086-y)
Supplement: Additional file 4: — Semi-structured questionnaire for an ethnobotanical analysis of parasitic plants in the Nepal Himalaya. (PDF 154 kb) [file 13002_2016_86_MOESM4_ESM.pdf]

Participant ID __ / __ / __ / ___ ___ Date: __ __/__ __/__ __
D D M M Y Y

Appendix IV: Semi-structured questionnaire for an ethnobotanical analysis of parasitic plants in the Nepal Himalaya.

An Ethnobotanical Analysis of Parasitic Plants in the Nepal Himalaya

Researcher: Alexander Robert O'Neill and Santosh Kumar Rana

Thank you for taking time to talk with me today. My purpose in talking with you is to learn about environmental change in Sikkim, including your interactions with wetlands, species utilization, and opinions on environmental change. You should know that the things you tell us here will remain confidential; we will not share the information you give us with your colleagues or any offical in a way that you will be able to be identified.

I freely and willingly consent to the following survey. Signature:

A. DEMOGRAPHICS

No	QUESTIONS	CODES
A1	What type of work do you do:	(1) Agro-pastoralist (2) NGO/ Non-Profit<br>(3) Education (8) Government Worker/Military<br>(4) Health/Medical (9) Trade/Technical<br>5) Other:________________________________
A2	What is your gender?	(1) Female (2) Male (3) Other _________
A3	What is your preferred language for the survey?	(1) English (2) Nepali (3) Other _________
A4	How old are you?	__________________years
A5	What is your household status?	(1) Single (3) Separated/Divorced (Skip 6)<br>(2) Married (4) Widowed (Skip 6)
A6	Do you live with your partner?	(0) No (1) Yes
A7	How many children do you have?	________ children
A8	How many people live in your house - share meals) (including you):	Adults: __________<br>Children under 15 years: ___________
A10	What is the highest grade of school you have completed?	A. School class __________<br>B. Professional Training:_________________ years:______<br>C. Post-secondary education (*type*): ________________<br>How many years: ______
A11	How long have you been working as a [__________________]:	_____years ______months **OR** Year started_______
A12	Where do you live?	___________________________________
A13	How long have you lived there?	_____years ______months **OR** Year started_______<br>**OR** Always (If always, Skip A14)
A14	Originally, where are you from?	City/Neighborhood: ___________________________________
A15	What languages do you speak?	(1) English (2) Hindi<br>(3) Nepali (4) Tibetan<br>(5) Other(s): ______________________
A16	What is your ethnicity?	(1) Nepali (2) Tamang(3) Gurung (4) Lepcha<br>(5) Chhetri (6) Bhutia (7) Sherpa (8) Subba/Limbu<br>(9) Other _______________

B. PLANT STATUS

No	QUESTIONS	CODES
B1	Have you seen this plant before?	(1) Yes (2) No
B2	Does this plant have a (vernacular) name?	Name:<br>Notes:
B3	Where can this plant be found?	(1) Forest (2) Field (3) Other<br>(A) Cultivated (B) Wild-harvested (C) Wild
B4	Does this plant grow alongside, on, or near other plants?<br>If so, which species?	(1) Alongside (2) On (3) Near (4) None<br>Notes:
B5	Does this plant influence the growth of the plants or plan communities where it is found?	(1) Promotes Growth (2) Hinders Growth<br>(3) No Effect(4) Unknown<br>Notes:
B6	How would you describe the growth habit of this plant?	
B7	Are there other plants in this area that have similar habits	(1) Yes (2) No (3) Unknown<br>Notes:
B8	Is this plant more common now or when you were a child	(1) More Common (2) Less Common<br>(3) No Change (4) Unknown
B9	If there has been a change, why do you think populations this plant have changed?	(1) Over-harvest (2) Deforestation<br>(3) Climate Changing<br>(4) Unknown (5) Other ___________<br>Note:
B10	Do people collect this plant?	(1) Yes (2) No (Unknown)
B11	If collected or cultivatd, has the amount of cultivation or collection changed from when you were a child to now? I this plant of conservation concern	(1) More (2) Less (3) Unchanged (4) Unknown<br>(A) Yes (B) No<br>Notes:

C. ETHNOBOTANICAL USES

No	QUESTIONS	CODES
C1	Is this plant used by local people	(1) Yes (2) No (3) Unknown
C2	If so, what types of uses:	Notes:
C2A	Food	When is it used?__________________<br>What parts are used? ______________<br>Do many people use it? ____________<br>How is it prepared? _______________<br>Notes:
C2B	Medicine	When is it used?__________________<br>What parts are used? ______________<br>Do many people use it? ____________<br>How is it prepared? _______________<br>Notes:
C3C	Material and/or Fodder	When is it used?__________________<br>What parts are used? ______________<br>Do many people use it? ____________<br>How is it prepared? _______________<br>Notes:
C3D	‘Cultural’ and Other	When is it used?__________________<br>What parts are used? ______________<br>Do many people use it? ____________<br>How is it prepared? _______________<br>Notes:
