# Supplementary material for: An ethnobotanical analysis of parasitic plants (Parijibi) in the Nepal Himalaya
Source: J Ethnobiol Ethnomed. 2016 Feb 24;12:14. doi: 10.1186/s13002-016-0086-y (PMC4765049; doi:10.1186/s13002-016-0086-y)
Supplement: Additional file 5: — Voucher records of collected parasitic plant species from Central and Eastern Nepal. (PDF 54 kb) [file 13002_2016_86_MOESM5_ESM.pdf]

Collection #	Collection Date	Species	Family	Altitude (m)	District
ARO1	11/12/13	*Cuscuta europaea* var. *indica*	Convolvulaceae	2500	Kaski
ARO2	11/20/13	*Cuscuta chinensis*	Convolvulaceae	1300	Kathmandu
ARO3	11/26/13	*Viscum articulatum* var. *liquidambaricolum*	Viscaceae	2000	Makwanpur
ARO4	12/04/13	*Pending Identification*	Loranthaceae	2200	Parbat
ARO5	04/14/14	*Loranthus odoratus*	Loranthaceae	2000	Ilam
ARO6	04/15/14	*Scurulla parasitica* var. *graciliflora*	Loranthaceae	2200	Ilam
ARO7	04/17/14	*Macrosolen cochinchinensis*	Loranthaceae	500	Jhapa
ARO8	04/21/14	*Cuscuta chinensis*	Convolvulaceae	375	Jhapa
ARO9	04/21/14	*Cuscuta reflexa* var. *reflexa*	Convolvulaceae	60	Jhapa
ARO10	04/22/14	*Cuscuta reflexa* var. *reflexa*	Convolvulaceae	80	Morang
ARO11	04/22/14	*Dendrophthoe pentandra*	Loranthaceae	80	Morang
ARO12	04/26/14	*Cuscuta reflexa* var. *reflexa*	Convolvulaceae	120	Bara
ARO13	04/26/14	*Cuscuta reflexa var. reflexa*	Convolvulaceae	120	Rautahat
ARO14	04/28/14	*Dendrophthoe falcata*	Loranthaceae	120	Rautahat
ARO15	04/30/14	*Viscum articulatum*	Viscaceae	2100	Makwanpur
ARO16	04/30/14	*Viscum articulatum* var. *articulatum*	Viscaceae	2100	Makwanpur
ARO17	04/30/14	*Viscum articulatum* var. *articulatum*	Viscacae	2000	Kathmandu
ARO18	04/30/14	*Cuscuta europea* var. *indica*	Convolvulaceae	1800	Kathmandu
ARO19	04/30/14	*Cuscuta reflexa* var. *reflexa*	Convolvulaceae	1500	Kathmandu
ARO20	04/30/14	*Viscum album*	Viscaceae	2100	Makwanpur
ARO21	04/30/14	*Helixanthera ligustrina*	Loranthaceae	1600	Makwanpur
ARO22	04/30/14	*Helixanthera ligustrina*	Loranthaceae	1300	Kathmandu
ARO23	04/30/14	*Scurulla elata*	Loranthaceae	1700	Makwanpur
ARO24	04/30/14	*Scurulla parasitica*	Loranthaceae	1800	Makwanpur
ARO25	04/30/14	*Taxillus vestitus*	Loranthaceae	1700	Makwanpur
ARO26	04/30/14	*Taxillus umbellifer*	Loranthacaeae	2800	Makwanpur
ARO26	04/30/14	*Scurulla elata*	Loranthaceae	1700	Makwanpur
ARO28	04/30/14	*Helixanthera parasitica*	Loranthaceae	1500	Makwanpur
ARO29	04/30/14	*Loranthus lamertianus*	Loranthaceae	1600	Makwanpur
ARO30	04/30/14	*Pending Identification*	Loranthaceae	1700	Makwanpur
ARO31	04/30/14	*Pending Identification*	Loranthacaeae	1600	Makwanpur
ARO32	04/30/14	*Scurulla pulverulenta*	Loranthacaeae	1600	Makwanpur
ARO33	04/30/14	*Pending Identification*	Loranthacaeae	1600	Makwanpur
ARO34	05/10/14	*Scurulla parasitica*	Loranthaceae	2200	Kaski
ARO35	05/10/14	*Scurulla pulverulenta*	Loranthaceae	2100	Kaski
ARO36	05/10/14	*Scurulla elata*	Loranthaceae	2100	Kaski
ARO37	05/15/14	*Scurulla pulverulenta*	Loranthaceae	1600	Nuwakot
ARO38	05/15/14	*Cuscuta reflexa var. reflexa*	Convolvulaceae	1600	Nuwakot
SKR39	06/25/13	*Monotropa uniflora*	Ericaceae	1500	Lalitpur
